# Effect of backbone conformation and its defects on electronic properties and assessment of the stabilizing role of π–π interactions in aryl substituted polysilylenes studied by DFT on deca[methyl(phenyl)silylene]s

**DOI:** 10.1186/s13065-016-0173-0

**Published:** 2016-05-05

**Authors:** Barbora Hanulikova, Ivo Kuritka, Pavel Urbanek

**Affiliations:** Centre of Polymer Systems, Tomas Bata University in Zlín, trida Tomase Bati 5678, 76001 Zlin, Czech Republic

**Keywords:** Density functional calculations, Kink, Methyl(phenyl)silylene, Stacking interaction, UV/Vis spectroscopy

## Abstract

**Background:**

Recent efforts in the field of mesoscale effects on the structure and properties of thin polymer films call to revival interest in conformational structure and defects of a polymer backbone which has a crucial influence on electronic properties of the material. Oligo[methyl(phenyl)silylene]s (OMPSi) as exemplary molecules were studied theoretically by DFT in the form of optimal decamers and conformationally disrupted decamers (with a kink).

**Results:**

We proved that *transoid* backbone conformation is true energy minimum and that a kink in the backbone causes significant hypsochromic shift of the absorption maximum (*λ*_*max*_), while backbone conformation altering from all-*eclipsed* to all-*anti* affects *λ*_*max*_ in the opposite way. π–π stacking was investigated qualitatively through optimal geometry of OMPSi and mutual position of their phenyls along the backbone and also quantitatively by an evaluation of molecular energies obtained from single point calculations with functionals, which treat the dispersion effect in the varying range of interaction.

**Conclusions:**

The kink was identified as a realistic element of the conformational structure that could be able to create a bend in a real aryl substituted polysilylene chain because it is stabilized by attractive π–π interactions between phenyl side groups.

**Graphical abstract:**
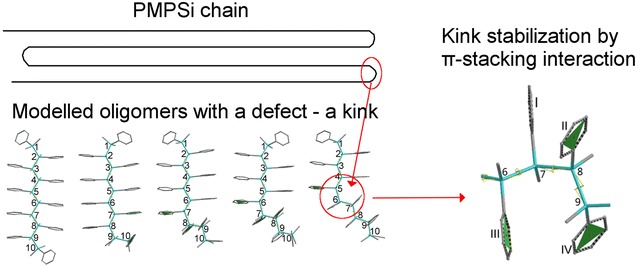
.

**Electronic supplementary material:**

The online version of this article (doi:10.1186/s13065-016-0173-0) contains supplementary material, which is available to authorized users.

## Background

Silicon (Si) polymers with -Si–Si- backbones carry delocalized σ-electrons as their sp^3^ orbital lobes can overlap [1, 2]. From this point of view, polysilylenes substantially differ from single-bonded carbon analogues (e.g. polyethylene, polystyrene), especially in the area of optoelectronic properties [3]. Electron delocalization origins in Si atoms arrangement and therefore it is highly dependent on the polysilylene secondary structure [4]. Maximum of σ-conjugation is related with all-*anti* backbone conformation, which can be found in dialkylsilylenes with small side groups, for instance poly(dimethylsilylene) (PDMSi) [5, 6]. On the other hand, poly[methyl(phenyl)silylene] (PMPSi) is arranged into helix due to presence of bulky phenyl (Ph) groups and with them related *deviant* or *transoid* backbone conformation [6–8]. Polysilylene chains are not single rod-like, they form random coil in solutions. Similarly in solid phase, the most of polysilylenes is semi-crystalline and contains regular as well as amorphous phase. Recent efforts in the field of mesoscale effects on structure and properties of thin polymer films made from both π- and σ-conjugated conductive polymers call to revival interest in conformational structure and defects of a polymer backbone which has crucial influence on electronic properties of the material. It has been already shown by different groups that polymer conformational order/disorder shows strong dependence on the thin film thickness in order of hundredths nm and results into non-trivial effects on optoelectronic properties in terms of segment conjugation length, luminescence, photovoltaic effect, exciton diffusion length [9–12] and fine bandgap electronic structure (density of deep states) [13, 14]. Obviously, the polymer structure itself and other typical polymer related properties [15–17] are influenced too. Hence, various bends of backbones are needed for the creation of regular or irregular arrangements. Such bend can be regarded as conformational defect because it disrupts regular σ-delocalization and therefore influences final polysilylene properties [18, 19]. This defect was defined as a *gauche*-kink in the backbone and described on oligo-DMSi_n_ (ODMSi) and oligo-MPSi_n_ (OMPSi) with n = 1–10 by density functional theory (DFT) in our previous work, where the kink influence on the electronic properties of oligosilylenes was confirmed [20]. The change has been clearly manifested in absorption spectra plots, where hypsochromic shift of the main absorption band had been detected. In addition, the shift is more strongly pronounced as the kink position altered closer the centre of a backbone. Another cause that is responsible for a rearrangement of the oligosilylene molecule can be identified as a charge carrier in its vicinity. From this reason, we have also investigated polaron quasiparticles of OMPSi_n_ with the introduced kink [21]. In that research, a significant change has emerged in a dependence of the spin density on the conformation of a backbone and its shift to more regular part of a Si–Si chain, i.e. a shift from the kink.

The p orbitals are distributed on the Ph rings in PMPSi and it seems reasonable that π–π interactions are employed during geometry arrangement and stabilization. This type of non-bonding interaction was described in detail by Hunter or Gung in 1990s, however the interaction has already been known since the first half of 20th century [22–24]. These interactions play an important role in stabilization of double helix of nucleic acids or other biologically active substances and they have been abundantly studied in these areas, e.g. Ref. [25–27]. The character of the interaction (i.e. whether the interaction is attractive or repulsive) depends on the mutual position of involved aromatic rings (on their distance and angle between planes). Several positions were described and defined; they are sandwich, parallel displaced (offset of rings), T-shape and edge-to-face arrangements. The first is representative of repulsive interactions as the p orbitals, which carry delocalized π-electrons, are oriented to each other. The rest evince attractive interaction, whose intensity is dependent on the particular ring offset [28, 29]. Recent research, e.g. review [30], has suggested not to use only the term π-stacking for a description of all non-bonding interactions between aromatic groups as it could be related predominantly to a rarely observed face-to-face arrangement and regarded as insufficient for expression of other offset positions.

Contemporary theoretical research often uses DFT and time dependent-DFT (TD-DFT) that has been established by Kohn and Sham [31–33] and Runge and Gross [34, 35], respectively. B3LYP (Becke-3-Lee-Young-Parr) model has been confirmed as suitable for calculations on silicon compounds [32, 36]. Its use for geometry optimization is indisputable and in many cases, it is as well as sufficient for calculation of spectral or thermal properties [37, 38]. However, B3LYP functional is not able to clearly distinguish energy changes related with non-bonding interactions which are better covered in density functionals involving dispersion term in their definition [39]. For π–π interaction energy evaluation are therefore usually used functionals such as M06 [40], ωB97X-D [41] or B3LYP-D [42], which are also able to characterise low- and long-range electron–electron interactions at various levels.

The present paper is another from the series of a computationally-led investigation of oligosilylenes and the purpose of this work is a determination of a mutual influence of silicon backbone conformation and conformational defect on the excitation properties of OMPSi_10_. Several constrained structures are here investigated to obtain a detailed and comprehensive view on the conformation issues as well as to confirm *deviant* or *transoid* conformation to be the global energy minimum. Description of π–π interactions of various conformations in the vicinity of the conformational defect is done through evaluation of phenyl angle-distance plot obtained from optimized geometries and molecular energy evaluation obtained from single point calculations with three different density functionals. We believe that results of this model study can be generalised and a useful lesson towards description of real polysilylene polymers can be learned from it.

## Experimental

PMPSi was obtained from Fluorochem Ltd. UK, GPC analysis revealed Mw = 27,600 g/mol and Mn = 8500 g/mol. Films for UV–Vis measurements were prepared by the spin coating method using spin coater Laurell WS-650-MZ-23NPP from the solution in toluene. Quartz glass was used as a substrate. The absorption spectrum was measured by Lambda 1050 UV/Vis/NIR spectrometer from Perkin Elmer.

## Computational methods

### Geometry optimization

Structures of OMPSi with ten repeated units (OMPSi_10_) were modelled with Spartan ´14 software (Wavefunction, Irvine, CA) [43]. Optimal geometry of decamer (later in this text designed as *10_opt*) was calculated with DFT on the level of B3LYP hybrid model and 6-31G(d) polarization basis set [44]. The backbone end atoms were capped with methyl groups and calculation was set in vacuum with no constrained bonds or angles. OMPSi_10_ with approximately *transoid* conformation was obtained as can be also found in our previous work [20]. This optimal structure was used for virtual preparation of other OMPSi_10_ analogues with a kink, which represents a conformational defect. The optimization of kinked decamers was performed with the same DFT model as described above and resulted in four OMPSi_10_ molecules. These structures differ in a position of the kink that adopted approximately *gauche* conformation. Geometry calculation of oligomers with a kink was described in detail in Ref. 11 these OMPSi_10_ were designed with *A*, *B*, *C* and *D* according to position of the kink and suffixed with *opt* as it is optimal structure with no constrained angles.

**Fig. 1:**
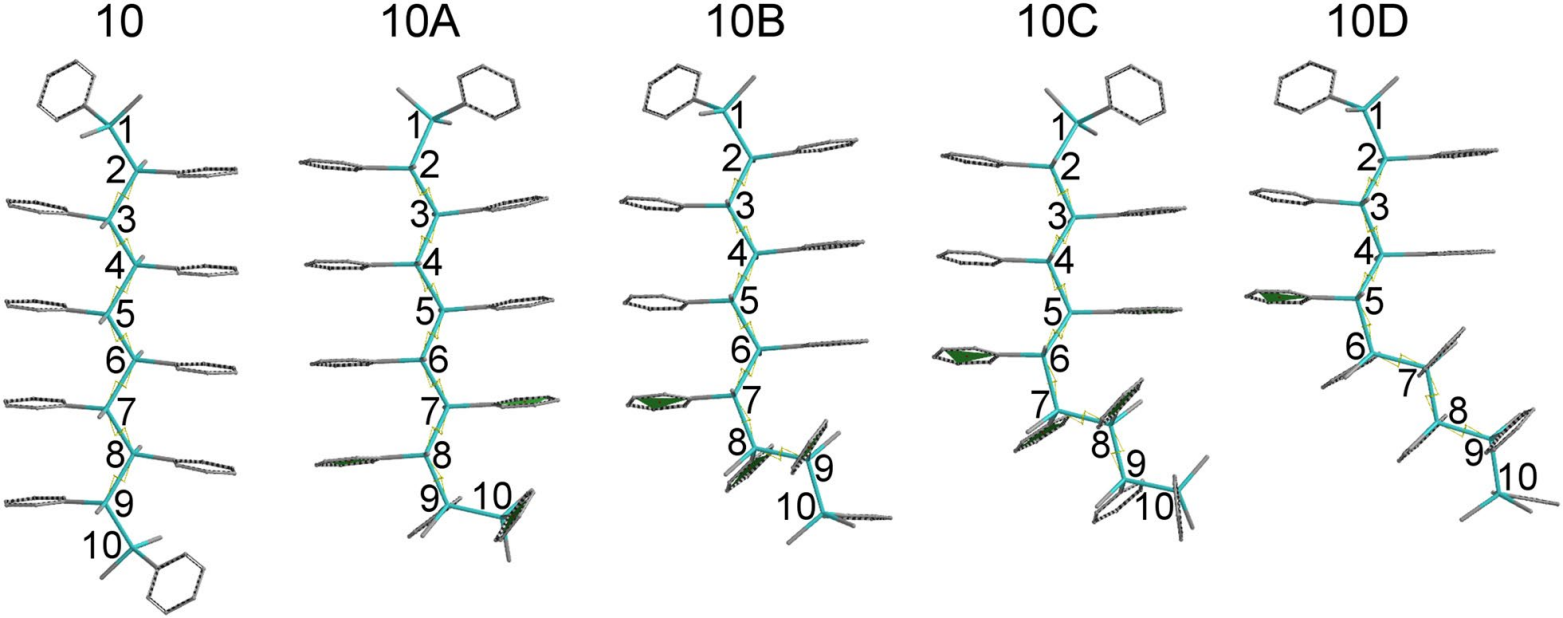
Geometries of OMPSi_10_ and designation of atoms and a kink position manifested on all-*anti* decamers (silicon atoms—*cyan backbone*, carbon atoms—*grey side groups*, hydrogen atoms—omitted)

More structurally specified molecules were modelled for the purpose of a description of an influence of the backbone conformation on the electronic structure of OMPSi_10_ with and without the kink, as well as for an assignment of the π–π interactions between phenyl groups. The dihedral angle of the kink was therefore constrained to 60° and all dihedral angles of a silicon backbone (ω) were set to 120°, 130°…180° and constrained as well. Moreover, a kink position is clearly given in Fig. 1. Geometry optimization was performed with DFT B3LYP/6-31G(d) in vacuum. From this calculation, seven structures of each decamer (*10*, *10A*–*10D*) with a backbone gradually coiled into helix were obtained. These structures are suffixed with *120*…*180* in their designation.

### Non-bonding interactions

Single point energy calculations were performed for all *10A…10D* OMPSi_10_ with M06 and ωB97X-D functionals, which are directly available in Spartan 14´ software. Although the absolute total energies obtained by these methods differ all three methods are known due to their low errors and variance of predicted values. Therefore, they can be used for prediction of trends and comparison of energy differences among series of conformers. The results calculated at higher levels of theory which includes non-bonding interactions were compared with molecular energy obtained with B3LYP which treats bonding interactions only. From the plots, which are given below, it was possible to determine the energy contribution to conformer stabilization caused by the weak phenyl– phenyl interactions because the Si backbone was constrained in all considered cases. The most energetically un-favourable conformation of the Si backbone with 120° dihedral angle was selected as the reference level. Hence, the contribution to the conformer stabilization due to σ-conjugation is predicted by B3LYP and the additional energy gain due to π-stacking is manifested as the difference between B3LYP and dispersion term including functionals.

### Absorption spectra

An investigation of electronic properties was done through examination of absorption spectra and excitation energies, including distribution of molecular orbitals and their percentage involvement into the process. These features and UV–Vis spectra were calculated with TD-DFT energy calculation in the excited state of OMPSi_10_. Functional, basis set and virtual environment of molecules were set as described in geometry optimization part. Optimal geometries in the excited state were not calculated due to excessive computer requirements.

## Results and discussion

### Backbone geometry and the kink

**Fig. 2:**
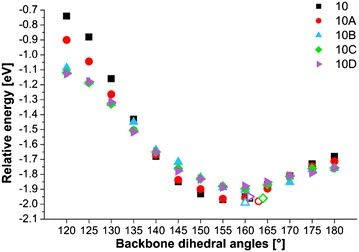
Energy profile of OMPSi_10_ with different backbone conformations (empty symbols: 10_opt…10D_opt)

Optimal geometry of ODMSi_10_ have already been determined in Ref. 11 and resulted in the helical backbone arrangement with dihedral angles corresponding to *transoid* conformation. An introduction of a kink has not influenced the rest of this arrangement in a significant extent. In the present work, more detailed conformational investigation have been done on several constrained OMPSi_10_ molecules, whose bond lengths are provided in Additional file 1. Figure 2 shows an energy dependence on the backbone conformation, which was set from all-*eclipsed* (120°) to all-*anti* (180°) arrangement. Relative energy on the y-axis was calculated by subtraction of—154849.74 eV (the calculated total energy of *10_120* decamer) from all other decamer energies. As can be seen, the energy minima are in all cases related with backbone dihedral angle 155° and 160° regardless the presence of the kink that is in agreement with optimal non-constrained OMPSi_10_. An approximately 5° difference can be attributed to 60°-locked kink dihedral angle in constrained structures.

### Molecular orbitals

**Fig. 3:**
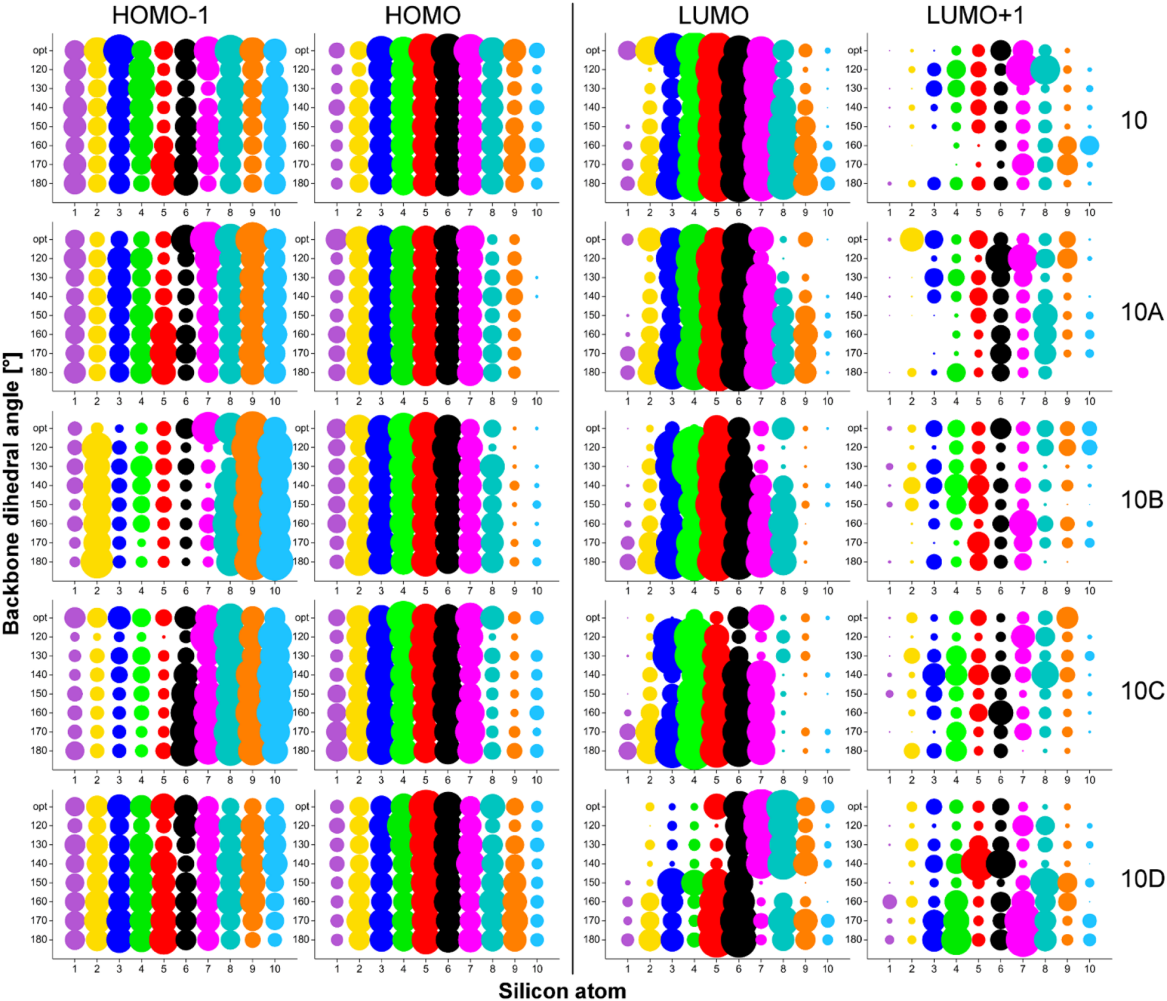
Kohn–Sham orbitals (H-1, H, L, L+1) distribution along Si backbone for all studied conformations of OMPSi_10_ (opt designates optimal geometry without constrained angles)

**Fig. 4:**
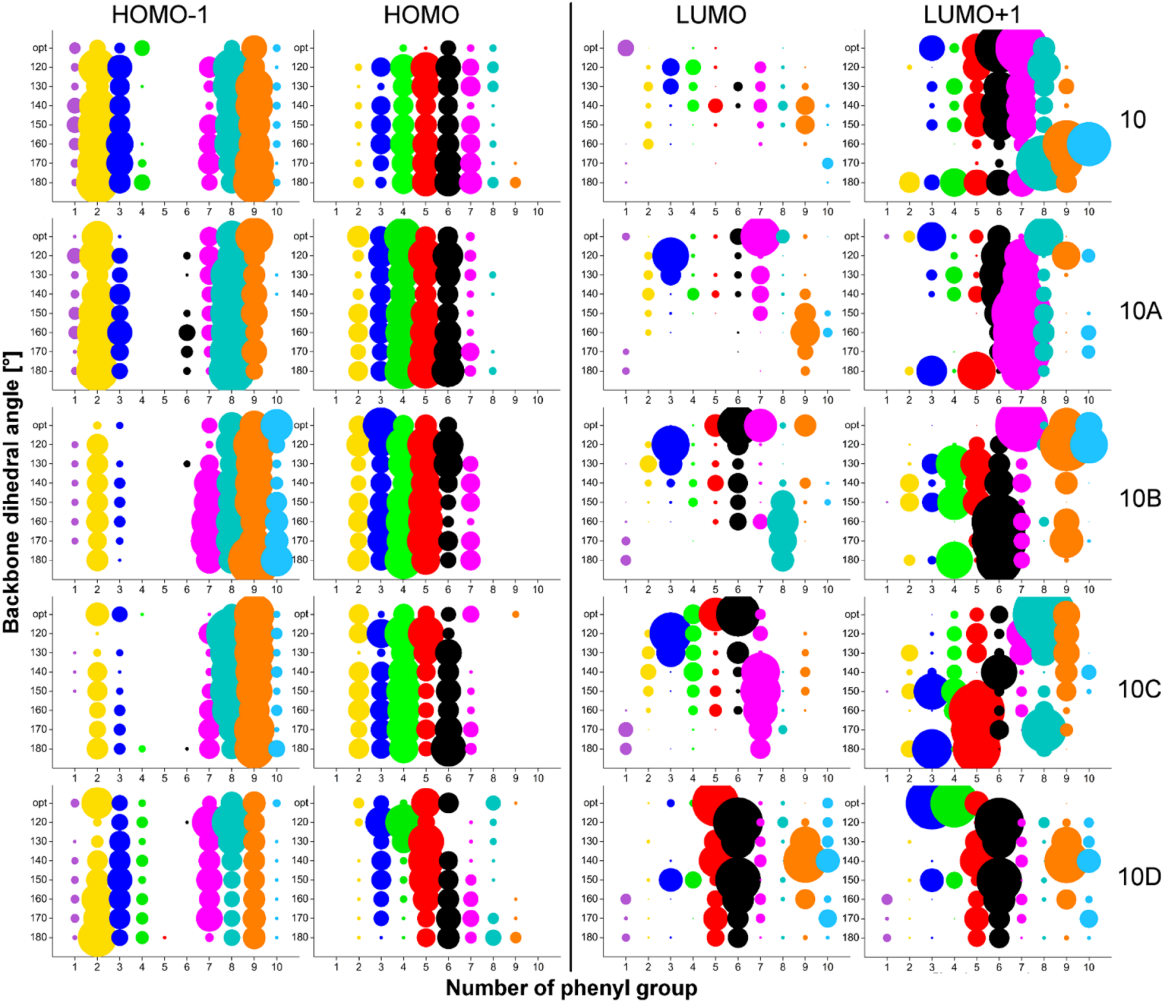
Kohn–Sham orbitals (H-1, H, L, L+1) distribution on phenyl groups for all studied conformations of OMPSi_10_ (opt designates optimal geometry without constrained angles)

Four molecular orbitals (MO) were investigated, namely HOMO-1 (H-1), HOMO (H), LUMO (L) and LUMO+1 (L+1), because these are involved in the excitation processes at the absorption maximum (described below). MO distributions along silicon backbone and Ph groups were plotted in the form of bubble graphs (Figs. 3, 4). The size of the bubble expresses a value of MO coefficients (*c*_*μi*_ in LCAO equation [45]) that were obtained from calculation output. Specifically, coefficients, whose absolute value is above the 0.05 threshold value were taken into account and at the same time coefficients related with particular atom (e.g. Si1) were summed. Analogous approach was applied to MO distribution on Ph groups but, in addition, MO coefficients related with the phenyl ring (i.e. six carbon atoms, while no density was transferred to hydrogen in any case) were summed. The size of the bubbles was graphically adjusted by multiplication to make the bubbles comfortably comparable. Thus, occupied and unoccupied MO coefficients were multiplied by 150 and 50, respectively.

Figure 3 depicts MO distribution along Si backbones for all studied decamers. As can be found, the main difference is observable between symmetric (*10* and *10D*) and asymmetric structures (*10A*, *10B* and *10C*). The symmetry is here given by the position of the kink and the fact that in *10A*, *10B* and 10*C* is backbone divided into two unequally length parts–segments. HOMOs-1 are basically delocalized along whole Si backbone in *10* and *D* molecules. *10A* OMPSi represents transition between symmetrical and asymmetrical structure as the kink is located in the very edge of a chain. HOMO-1 of *10A* molecules is thus distributed almost symmetrically along the backbone, however a slight shift to a kink part is already observable. This shift of HOMO-1 towards the kink and its localization on the shorter segment is clearly visible in *10B* and *10C* decamers. Similarly as HOMOs-1, HOMOs of *10* and *10D* decamers are distributed equally along Si–Si bonds and maximal values of *c*_*μi*_ can be found on central Si atoms. On the other hand, in *10A*–*10C*, HOMO orbitals are shifted from the kink part and maxima are kept in the middle of chains on Si4–Si6. The effect of a kink introduction on HOMOs seems to be of lower intensity than in case of HOMO-1 but this is only a semblance perception of the graph because the delocalization length over the longer segment is just longer, naturally. An influence of different ω is in both cases of HOMOs-1 and HOMOs distribution negligible.

Unoccupied MOs are more dependent on the overall backbone arrangement. As can be further seen in Fig. 3, LUMOs of all *10* structures are distributed along chain with higher values of coefficients in the central parts. This central gathering is particularly observable in *10_120*. *10A*s, *10B*s and *10C*s carry LUMOs in longer parts of Si chain and this shift from kink part is especially observable in conformers with ω = 120°. *10D*s are the most influenced structures by ω value. Since the kink is located in the middle, the preference for LUMO delocalization is determined by the values of backbone dihedral angles. *10D_120–150* have LUMO orbitals located rather on one half of backbone and in *10D***_***160–180*, the delocalization is again symmetrical almost along the whole chain. LUMO+1 orbitals are delocalized on Ph parts (described below) and they are presented on Si backbone in much less extent. There is no simple trend that could easily sum the kink and conformation influence up. Increasing ω causes variable shifts including opposite trends in dependence on the kink position. Images of all these Kohn–Sham orbitals that graphically express the bubble graphs are given in Additional file 2: Figures S1–S4.

Figure 4 reflects MO distribution on Ph rings attached along backbone. Rings are numbered according to the position of Si atom to which the ring is attached (e.g. a bubble on a position (1; 120) corresponds to sum of MO coefficients from six carbons that form the Ph ring attached to Si1 in conformer 120°). As can be observed, MO on Ph rings are much more localized in comparison with MO along Si backbone. HOMOs-1 are distributed on the edge phenyls while the phenyl groups attached to central Si5 and Si6 atoms remain practically not involved into the orbital delocalization. In no-kink structures of *10* delocalization is symmetrical and this characteristic splitting is also kept in other molecules but with a lesser extent of symmetry. HOMOs-1 of *10A–10C* are preferentially localized on Ph groups adjacent to the kink and to the shorter segment of the decamer. In the case of *10D* molecules, the symmetry is again restored, although to a lesser extent than in *10* oligomers. On the other hand, HOMOs seem to appear rather on the central Ph rings and on the longer segment up to Si1 (cases *A, B, C*). The more is the kink close to the centre of the decamer, the more these HOMOs are squeezed to that longer segment and kink-attached Ph groups are more involved in HOMO, which is an opposite effect than manifested for HOMOs-1. The population density of HOMOs on the two segments of symmetric *10D* cases depends on ω. The optimized structure has the HOMO distributed more on the silicon chain than any other structure under investigation. The tested geometries have bigger population density located on phenyl groups. With increasing angle from 120° to 180°, the density becomes less symmetric and shifts from left to right (from lower number positions to higher number positions) having thus always quite densely populated Ph5 and Ph6. It must be stressed out that Ph rings adjacent to Si atoms forming the kink are involved in the MO delocalization. In both cases of HOMOs-1 and HOMOs, the overall distribution of occupied MOs is influenced by the presence of the kink and conformation of the backbone however it does not mean that Ph rings adjacent to the kink Si atoms are excluded from the delocalization.

It can be stated that LUMOs are present on Ph rings rarely. There are only a few Ph groups that carry LUMO in the considerable extent. Seemingly, the Ph group attributable portion of LUMOs in optimal conformations of OMPSi_10_ is located on that Ph group from the kink part in all kinked structures which is attached to the Si atom closer to the longer segment or in other cases the LUMO density is located on the two Ph groups attached to those two Si from the kink with lower position numbers, which means that these MOs are shifted from Ph7 to Ph5. *10D* OMPSi_10_ carry LUMOs particularly on Ph5 and Ph6 irrespective of the dihedral angle of the backbone with exception of some population density located to the Ph9 for angles 130° and 140°. On the contrary, LUMO+1 delocalization is strongly related with Ph rings when compared with Si backbone orbitals. 180° conformations are the most symmetrical cases, which are affected by the kink presence. Generally, LUMOs+1 are significantly distributed on one or two Ph rings according to a kink position and backbone conformation. The ω has the largest effect on the distribution of LUMOs+1 among tested parameters as it evidently prevail over the importance of the kink position. This influence scatters the manifestation of kink-caused trends and makes the results less readable than in all previous cases. Images of Kohn–Sham orbitals distributed along phenyl rings are appended in the Additional file 2.

### Excitation properties

**Fig. 5:**
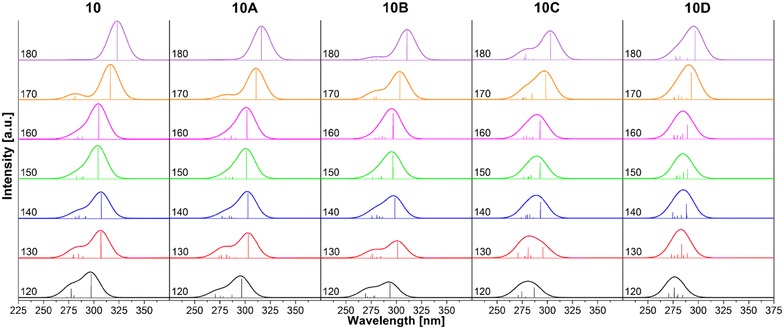
UV–Vis spectra of all studied OMPSi_10_ calculated with TD-DFT B3LYP/6-31G*

TD-DFT approach was used to calculate UV–Vis spectra and related excitation properties. Figure 5 depicts a palette of absorption spectra corresponding to every considered OMPSi_10_ conformer. There are also line spectral bands that are helpful for determination and comparison of transition intensities. Graphical information are supplemented by Table 1, where the data describing excitation at the highest wavelength (λ_max_) are given. Comprehensive characterization of all calculated transitions is given in Additional file 3: Table S1.

**Table 1:** Summary of excitation process at *λ*_*max*_ for all OMPSi_10_

	ω[°]	120	130	140	150	160	170	180
10	E [eV]	4.1699	4.0404	4.0366	4.0773	4.0690	3.9203	3.8389
	λ [nm]	297.33	306.86	307.15	304.08	304.70	316.26	322.96
	F	0.9208	1.0192	0.9681	1.2094	1.3080	1.3262	1.4727
	TT	H → L	H → L	H → L	H → L	H → L	H → L	H → L
	Amp.	0.9708	0.9683	0.9671	0.9615	0.9746	0.9783	0.9809
	P [%]	94	94	94	92	95	96	96
10A	E [eV]	4.1817	4.0899	4.0966	4.1151	4.1085	3.9892	3.9221
	λ [nm]	296.49	303.15	302.65	301.29	301.77	310.80	316.20
	F	0.7367	0.9185	0.9709	1.0888	1.1393	1.1801	1.2672
	TT	H → L	H → L	H → L	H → L	H → L	H → L	H → L
						H → L+4		
	Amp.	0.9698	0.9672	0.9624	0.9545	0.9467	0.9755	0.9801
						−0.2136		
	P [%]	94	94	93	91	90	90	96
						–		
10B	E [eV]	4.2219	4.1181	4.1520	4.1795	4.1771	4.0856	3.9927
	λ [nm]	293.67	301.07	298.42	296.65	296.82	303.47	310.53
	F	0.5475	0.6299	0.7831	0.9174	1.0123	1.0427	1.1435
	TT	H → L	H → L	H → L	H → L	H → L	H → L	H → L
				H → L+1				
	Amp.	0.9630	0.9762	0.9411	0.9509	0.9270	0.9634	0.9756
				0.2428				
	P [%]	93	95	89	90	86	93	95
				–				
10C	E [eV]	4.3223	4.1951	4.2310	4.2375	4.2371	4.1556	4.0906
	λ [nm]	286.85	295.54	293.04	292.59	292.61	298.35	303.09
	F	0.3999	0.4321	0.6311	0.6312	0.7266	0.9813	1.0675
	TT	H → L	H → L	H → L	H → L	H → L	H → L	H → L
		H → L+3	H → L+1	H → L+1				
	Amp.	0.9155	0.9194	0.8261	0.9330	0.9385	0.9607	0.9684
		−0.2189	0.2450	-0.4610				
	P [%]	84	85	68	87	88	92	94
		–	–	21				
10D	E [eV]	4.3609	4.2885	4.2973	4.2880	4.2879	4.2313	4.1795
	λ [nm]	284.30	289.11	288.52	289.14	289.15	293.02	296.67
	F	0.1020	0.1895	0.1880	0.3833	0.5503	1.0527	1.1367
	TT	H-1 → L	H → L	H → L	H → L	H → L	H → L	H → L
		H → L	H → L+1	H → L+1				
			–	H → L+2				
	Amp.	0.2525	0.8842	−0.3215	0.9266	0.9117	0.9305	0.9439
		0.9225	0.2587	0.6534				
		–	–	−0.5969				
	P [%]	85	78	10	86	83	87	89
		–	–	43				
		–	–	26				

*ω* dihedral angle, *E* excitation energy, *λ* wavelength of excitation, *f* strength, *TT* type of transition, *Amp* amplitude, *P* percentage of allowed transition

**Fig. 6:**
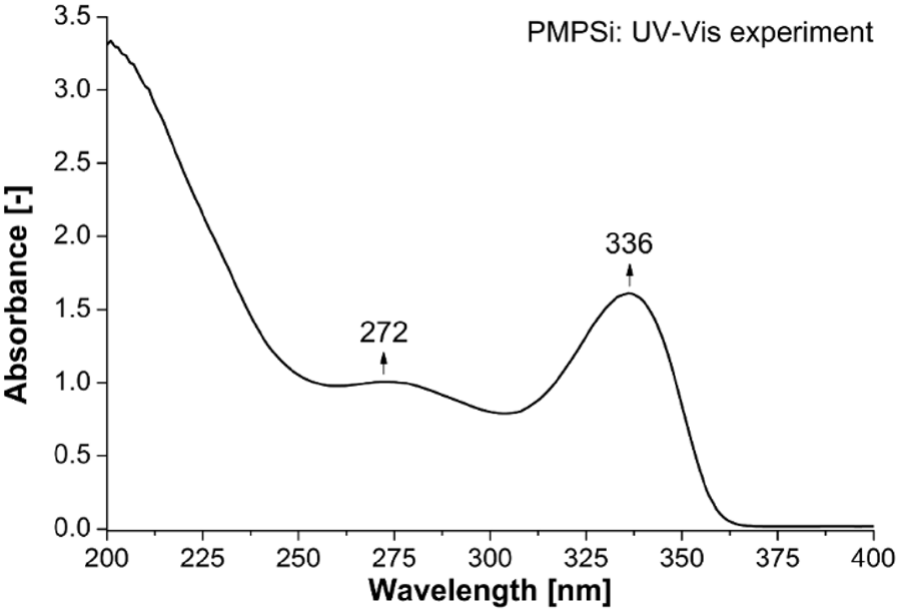
Experimental UV-Vis spectrum of PMPSi

As can be deduced, the maximum wavelength absorption is, in the vast majority, at the same time the most intensive one. The main character of this transition is σ → σ* occurring between Si orbitals H → L, in some cases H-1 → L or H → L+1 and exceptionally H → L+4 and L + 6. Further, in 120°, 130°, 170°and 180° analogues, second absorption band is clearly seen. The transition is from H or H-1 to higher unoccupied MO, which are located on phenyl rings. This indicates σ → π* transition from Si atoms to Ph groups. This transition is in literature often assigned as π–π* [46], however we propose in accordance with our theoretical results that this band better corresponds to σ → π* transition. π–π* transition is probably of higher energy and it is located close to 200 nm. The band below 200 nm is partially observable in experimental spectrum of PMPSi in Fig. 6.

Calculated wavelengths are compared with experimentally measured UV–Vis spectra of PMPSi which is shown in Fig. 6. The spectrum contains peaks in the UV part of the spectrum since no sign of the absorption is manifested in Vis area. There are two absorption bands in UV range which can be also identified in calculated spectra, rather of coiled decamers with a non-centrally placed kink. This indicates that the real PMPSi backbone is not planar and straighten but it is rather in helical arrangement. This is in agreement with our optimal geometries with lowest potential energy. On the other hand, two band are observable in *180_B* and *180_C* OMPSi_10_ too. In these cases, the kink probably serves as a “helical mimic” structural element which delivers twisted-like conformation to the oligomer that causes similar spectral behaviour, which has been described for helical backbones. The difference between experiment and theory is, of course, observable predominantly due to comparison of experimental spectrum of polymer and theoretical spectrum of isolated decamer and therefore calculated spectral bands are energetically overestimated about several tenths of eV which is in accord with expectable eventual solvation effect of toluene. However, this drawback would not destroy the main trends referring to conformation and electronic behaviour of polysilylene and addition of solvent force field terms to calculations can neither significantly improve our virtual experiment nor clarify the role of phenyl–phenyl group interaction.

It is important to note that no states in the bandgap are formed by the investigated conformational defects, which means that no peaks are present in the Vis area of the absorption spectrum. This is in accordance with state-of-the-art interpretation of origin of such features which are normally manifested in luminescence spectra only [11].

**Fig. 7:**
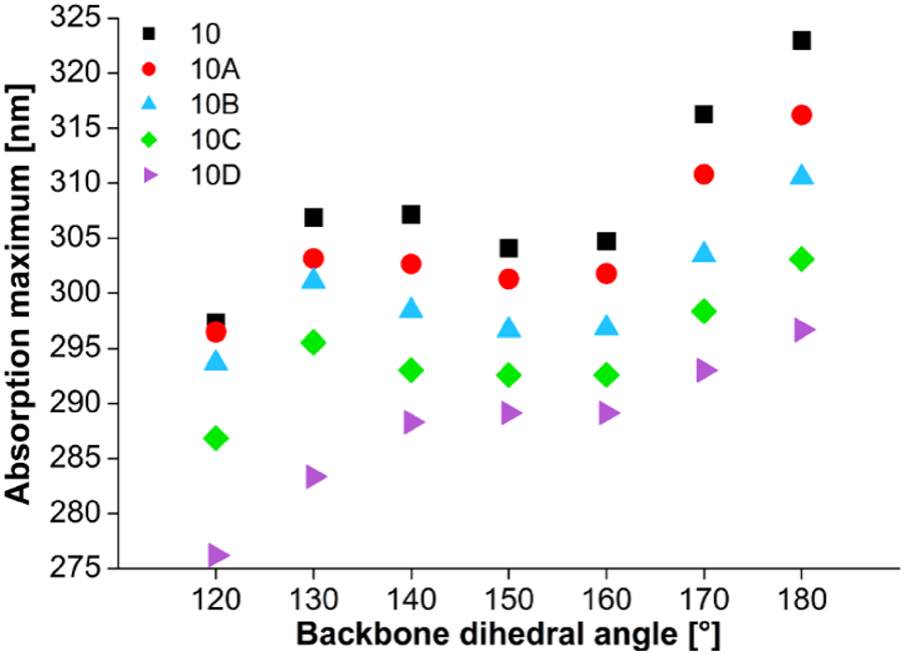
Dependence of the absorption maximum wavelength on backbone dihedral angle

Figure 7 provides another view on a dependence of *λ*_*max*_ on the backbone conformation. It is unambiguous that *λ*_*max*_ shifts to longer UV wavelengths as ω is higher and thus as backbone conformation reaches planar all-*anti* arrangement. All structures with ω = 150°, 160° evince decrease of *λ*_*max*_ or in case of *10D* a stabilization of *λ*_*max*_ value. These conformers are also the most energetically stable as was discussed above (see again Fig. 2). Following change in ω causes another and substantial growth of *λ*_*max*_ that reaches maximum for ω = 180°. There is also obvious that presence and position of the kink significantly influences a value of *λ*_*max*_. As can be seen, 10 and 10A decamers are the most similar and change in *λ*_*max*_ for *10A* is not so large. On the other hand, difference between *10* and *10C* molecules is in some conformations around 10 nm and between *10* and *10D* even 25 nm. This proves that conformational defect has essential effect on excitation wavelength that is a crucial factor of UV–Vis absorbing substances.

### π–π interactions between phenyl side groups

**Fig. 8:**
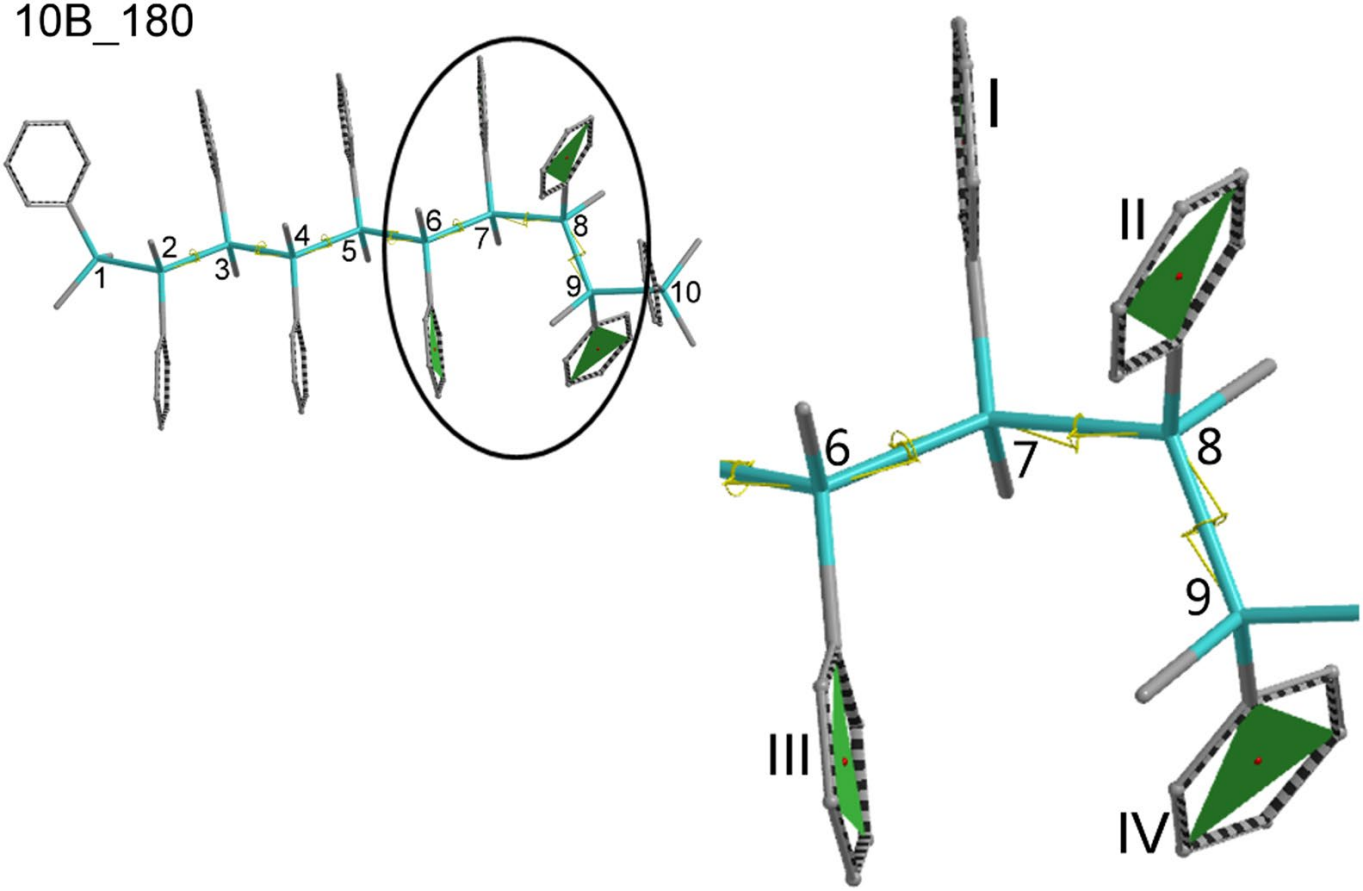
Designation of phenyl planes regardless the position of the kink shown on example molecule 10B_180

Studied OMPSi_10_ structures are example of the system, which can interact through p orbitals occupied by π-electrons. Figure 8 contains a structure of *10B_180* molecule with a detailed image of a kink part and a designation of phenyl planes, which are attached right on four Si atoms which form the kink. Numbers of planes are valid for all structures regardless the position of the kink. The kink has set exact arrangement of *gauche* in all cases and since the backbone is also geometrically defined Ph groups could have therefore adopted various optimal positions.

**Fig. 9:**
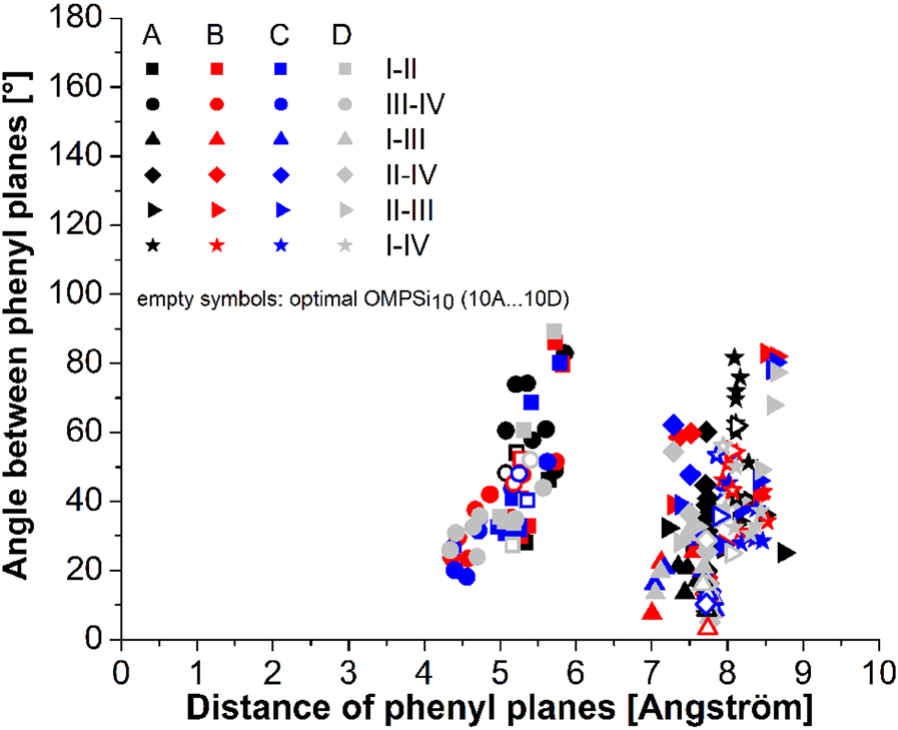
Plot of positions of phenyl groups located on the kink Si atoms. Each *symbol* in the legend table involves seven conformers (120–180) which are not graphically distinguished in the *plot*

**Fig. 10:**
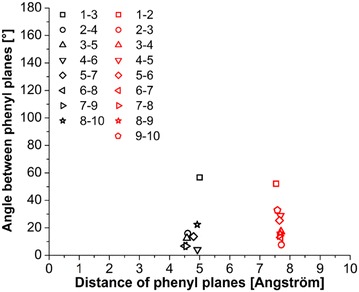
Plot of positions of all pairs of phenyl groups located along backbone of 10_opt structure

A qualitative evaluation of π–π interactions is done through definition of mutual positions of the phenyl groups obtained solely from geometry optimization procedure. A plane on each involved Ph have been determined with three points (three phenyl C atoms) and a central point was defined as a point in the middle of a line, which links two opposite phenyl C atoms. Thus, Fig. 9 depicts an angle-distance dependence of these Ph groups. An angle was measured between two Ph planes and a distance was measured between two plane central points. In total, six pairs of phenyl groups have been investigated for each *A–D* and *120–180* decamer. As can be seen from the plot, there are two distinct clouds of points clearly separated by an approximately 1 Å wide gap virtually centred at 6.5 Å. According to Ref. 15, attractive π–π interactions can be found between planes I-II and planes III-IV, whose mutual positions are in the graph area of 4–6 Å and 10–90°. This indicates that a kinked arrangement of the chain could be stabilized by these interactions and therefore this type of bending is possible to consider as a folding contribution element in the real polymer backbones. These constructive interactions may also contribute to the localization of MOs on Ph rings attached to Si atoms forming the kinks. Another cluster of points is located in the area of 7-9 Å and 0-90° and it can be stated that the vast majority of plane pair I–III, II–IV, II–III and I–IV is in a further distance then that which is suitable for any kind of π–π stacking interactions. Further, Fig. 10 is similar representation of π–π interactions for *10_opt* structure, which were here investigated along the whole chain. For this purpose, phenyl rings were numbered from 1 to 10. As can be seen, there are also two groups of points. The first cluster (at approx. 4–5 Å) belongs to measurements of angle-distance dependence of Ph pair which are next to each other (on the same side of a chain) along the backbone. Ph groups are designed in this graph with numbers corresponding to a Si atom they are attached on. These interactions can be regarded as attractive and thus the helical arrangement of a backbone is favourable. The latter cluster (at approx. 7–8 Å), which involves interactions of adjacent Ph (in zig-zag way), is again beyond the marginal distance suitable for π-stacking.

**Fig. 11:**
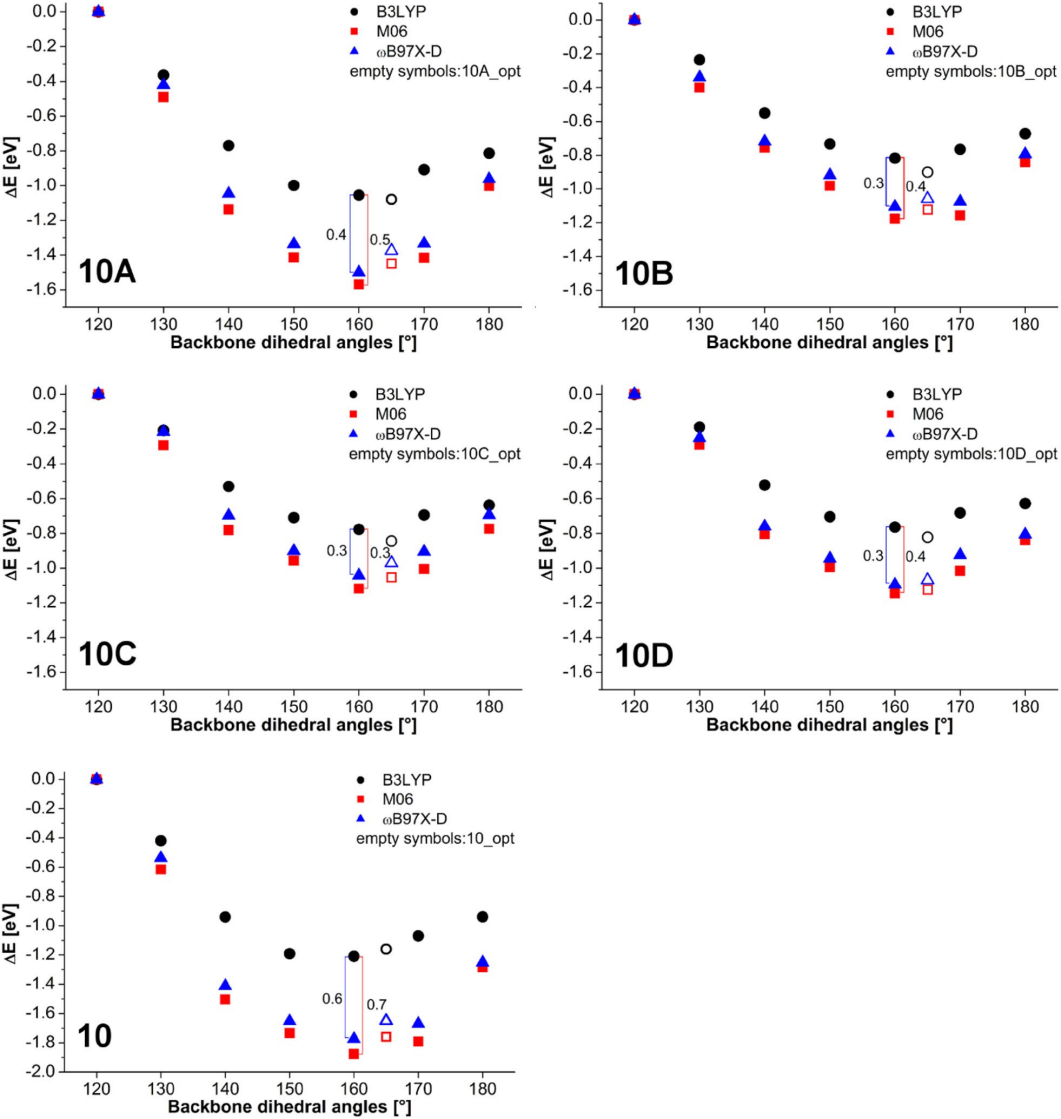
Single point calculations with different functionals concerning non-bonding phenyl interactions

Figure 11 depicts the energy profile that is related with a phenyl rings rearrangement on model molecules with different backbone geometry (kink position and dihedral angle). The plots were obtained from single point calculations and comparison of B3LYP and dispersion containing functionals M06 and ωB97X-D. Raw energy data are displayed in Additional file 4: Table S2, however y-axis in Fig. 11 expresses the energy difference (ΔE) in eV between OMPSi with the kink in the same position (i.e. *10A*, *10B*, *10C, 10D*) and at the same time the zero value corresponds to conformers with ω = 120**°**. Calculated B3LYP energies reflect the situation where long distance phenyl interactions are not involved. These curves describe only energy dependence on the dihedral angle and they can be interpreted as the contribution of σ-conjugation to the chain stability which increases as the geometry approaches closer to the ideal value for ω which is approximately 165°. Therefore the B3LYP energies can be considered as reference values. On the other hand, M06 and ωB97X-D energies do involve low-range and long-range electron–electron interactions, respectively. Since backbone dihedral angles were constrained in all cases, these energies are directly related to phenyl rings energy contribution to their mutual interactions. Molecules which are conformationally more convenient for π–π stacking thus have lower energy. The kink-less geometry (*10*) shows the highest stabilization contribution 0.6 eV which is consistent with its most relaxed geometry and ideal-likeness of molecule conformation. According to our results, π–π interactions are employed gradually with an increasing ω and they reach maximum in OMPSi which have ω constrained to 160° and 170° and then their strength again decreases. The additional contribution of these interactions is marked in graphs by vertical line segments with indicated difference in eV. These conformations are tightly close to optimal geometries obtained without any backbone constrain (also displayed in Fig. 11) and therefore it is highly probable that kink stabilization by non-bonding interactions can be expected in PMPSi chains. In other words, the kink formation disturbs slightly the stabilization effect of π-interactions, however it does not vanish totally and still keeps a reasonable contribution. The maximal difference between B3LYP and M06 and B3LYP and ωB97X-D for molecules *A*–*D* is 0.4 eV and 0.3 eV in average, respectively.

## Conclusions

OMPSi_10_ served as model systems for the DFT study of overall backbone conformation with conformational defect (a kink), its influence on electronic properties and an investigation of the kink stability provided by π–π stacking interactions. Helical backbones with Si–Si–Si–Si angles equal to 150° and 160° have been determined as the most stable backbone arrangements. Conformations have been treated from 120° to 180° and together with the kink they significantly affect the distribution of Kohn–Sham orbitals along both Si backbone and Ph side groups. HOMO-1 orbitals are distributed along the backbone, while LUMO+1 orbitals are strictly kept on Ph groups. Further, HOMO and LUMO densities can be found delocalized over the whole molecule.

The main calculated absorption transition is assigned as σ–σ* and located at around 310 nm, in experimental UV–Vis spectrum at 336 nm. Second transition around 275 nm is probably of σ–π* character despite traditional assignment to π–π*. We presume that π–π* corresponds energetically to lower wavelengths below 200 nm. However, optimal geometries of excited states have not been successfully calculated due to too demanding computer requirements and at the same time these calculations could be a topic of the next research leading to specification of excitation transitions.

The analysis of angle-distance dependence between Ph planes determined from optimized geometries has revealed that even the molecule with a kink is stabilized by positive interactive mode of π–π stacking between pairs of Ph groups. Conformations with backbone dihedral angle of 160° are the most convenient for phenyl interactions, which was concluded from energy investigation with B3LYP, M06 and ωB97X-D models. There can be distinguished two principally different contributions to the PMPSi backbone geometry. First, it is the previously well-known σ-conjugation effect that has been estimated in order of approximately 1.2 eV Next, the long range π–π interaction contribution was found to be about 0.6 eV for linear chain and about 0.3–0.4 eV for kink defect containing chains. Since 160° conformation is close to the optimal geometry of OMPSi without constrained parts, it can be stated that the kink type of conformational defect is viable in real PMPSi chains.

## Additional files

10.1186/s13065-016-0173-0 Contains raw data of bond lengths of all OPMSi_10_ structures as obtained from Spartan 14´ output. The following additional data are available with the online version of this paper.
10.1186/s13065-016-0173-0 Contains four sets of figures of Kohn-Sham orbitals for all studied deca[methyl(phenyl)silylene]s in various backbone conformations and with an introduced kink in the chain. Backbone dihedral angles altered from 120° to 180° and the kink position altered from the edge of chain (A) to the centre part of chain (D).
10.1186/s13065-016-0173-0 Contains a table with information on excitation process of all studied deca[methyl(phenyl)silylene]s in various backbone conformations and with an introduced kink in the chain. Backbone dihedral angles altered from 120° to 180° and the kink position altered from the edge of chain (A) to the centre part of chain (D).
10.1186/s13065-016-0173-0 Molecular energies calculated for all studied deca[methyl(phenyl)silylene]s obtained with B3LYP, M06 and ωB97X-D functionals.
