# Supplementary material for: Effect of backbone conformation and its defects on electronic properties and assessment of the stabilizing role of π–π interactions in aryl substituted polysilylenes studied by DFT on deca[methyl(phenyl)silylene]s
Source: Chem Cent J. 2016 May 5;10:28. doi: 10.1186/s13065-016-0173-0 (PMC4858925; doi:10.1186/s13065-016-0173-0)
Supplement: Supplementary file 2 — 10.1186/s13065-016-0173-0 Contains four sets of figures of Kohn-Sham orbitals for all studied deca[methyl(phenyl)silylene]s in various backbone conformations and with an introduced kink in the chain. Backbone dihedral angles altered from 120° to 180° and the kink position altered from the edge of chain (A) to the centre part of chain (D). [file 13065_2016_173_MOESM2_ESM.pdf]

Effect of backbone conformation and its defects on electronic properties and assessment of the stabilizing role of π-π interactions in aryl substituted polysilylenes studied by DFT on deca[methyl(phenyl)silylene]s

Barbora Hanulikova, Ivo Kuritka, Pavel Urbanek*

Centre of Polymer Systems, Tomas Bata University in Zlín, trida Tomase Bati 5678, Zlin, 76001, Czech Republic

*Corresponding author, email: hanulikova@cps.utb.cz

Additional data file 2

Images of Kohn-Sham orbitals for all studied deca[methyl(phenyl)silylene]s in various backbone conformations and with an introduced kink in the chain. Backbone dihedral angles altered from **120**° to **180**° and the kink position altered from the edge of chain (**A**) to the centre part of chain (**D**).

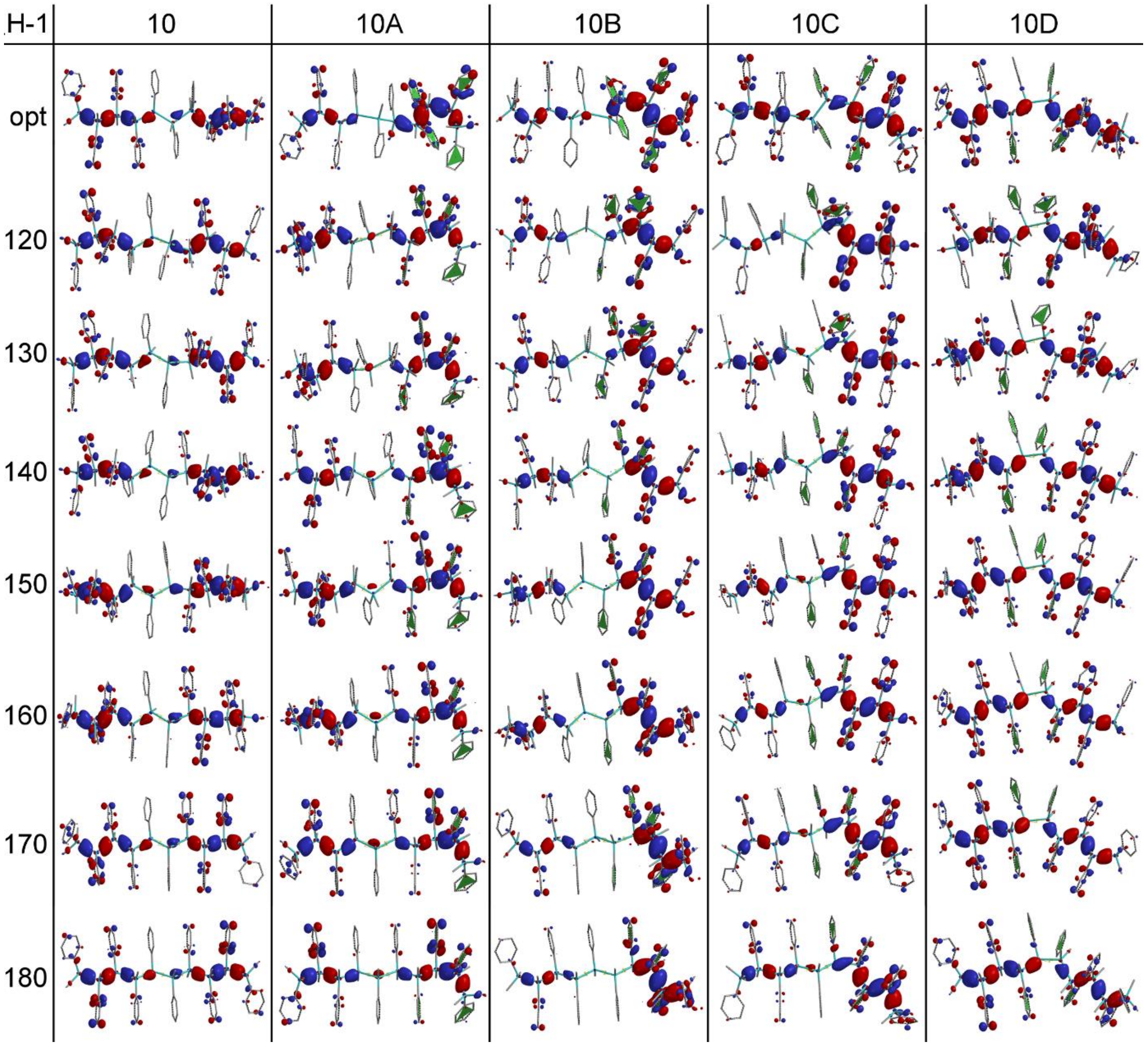

Figure S1. Kohn-Sham orbitals distribution: HOMO-1.

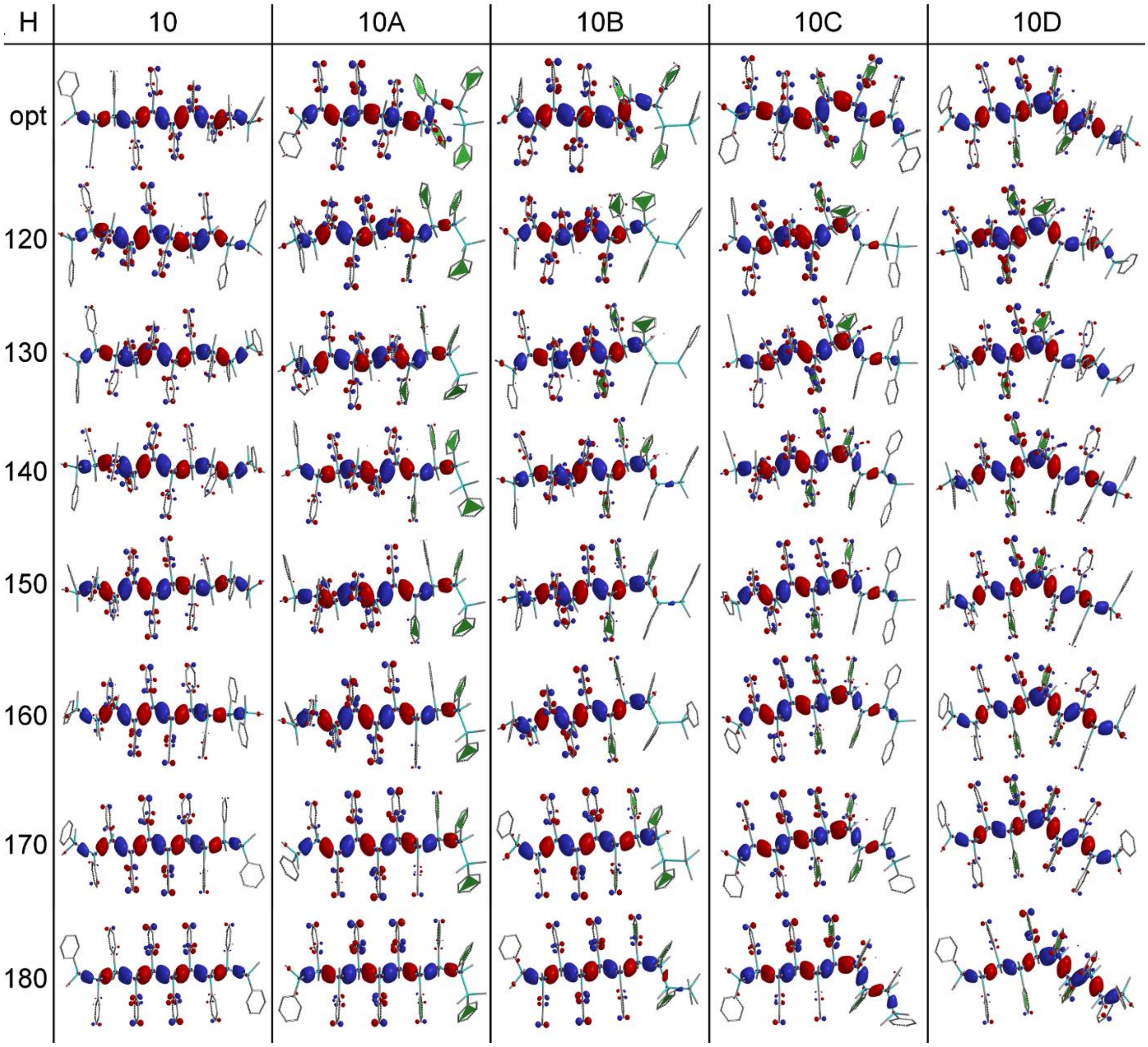

Figure S2. Kohn-Sham orbitals distribution: HOMO.

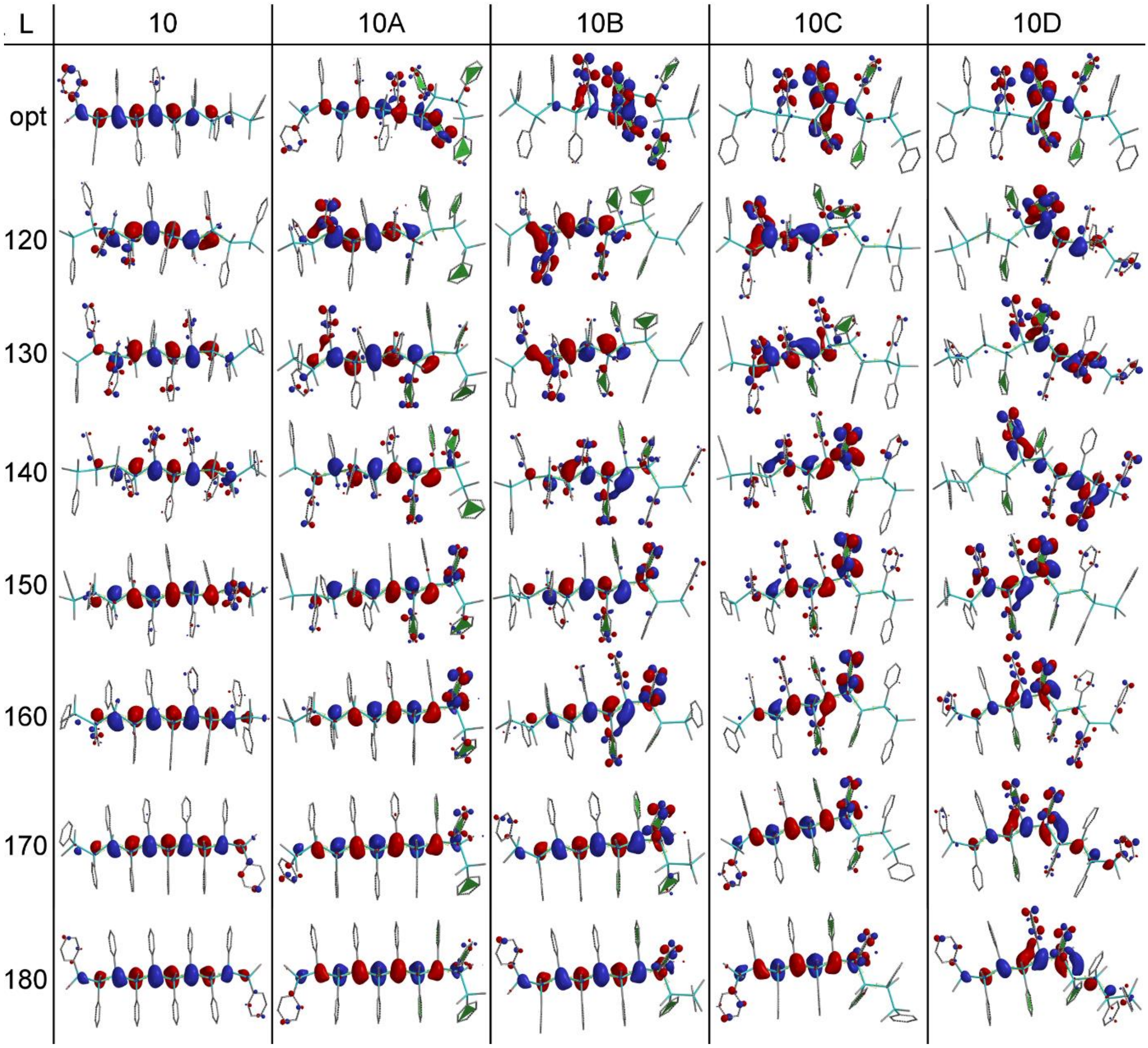

Figure S3. Kohn-Sham orbitals distribution: LUMO.

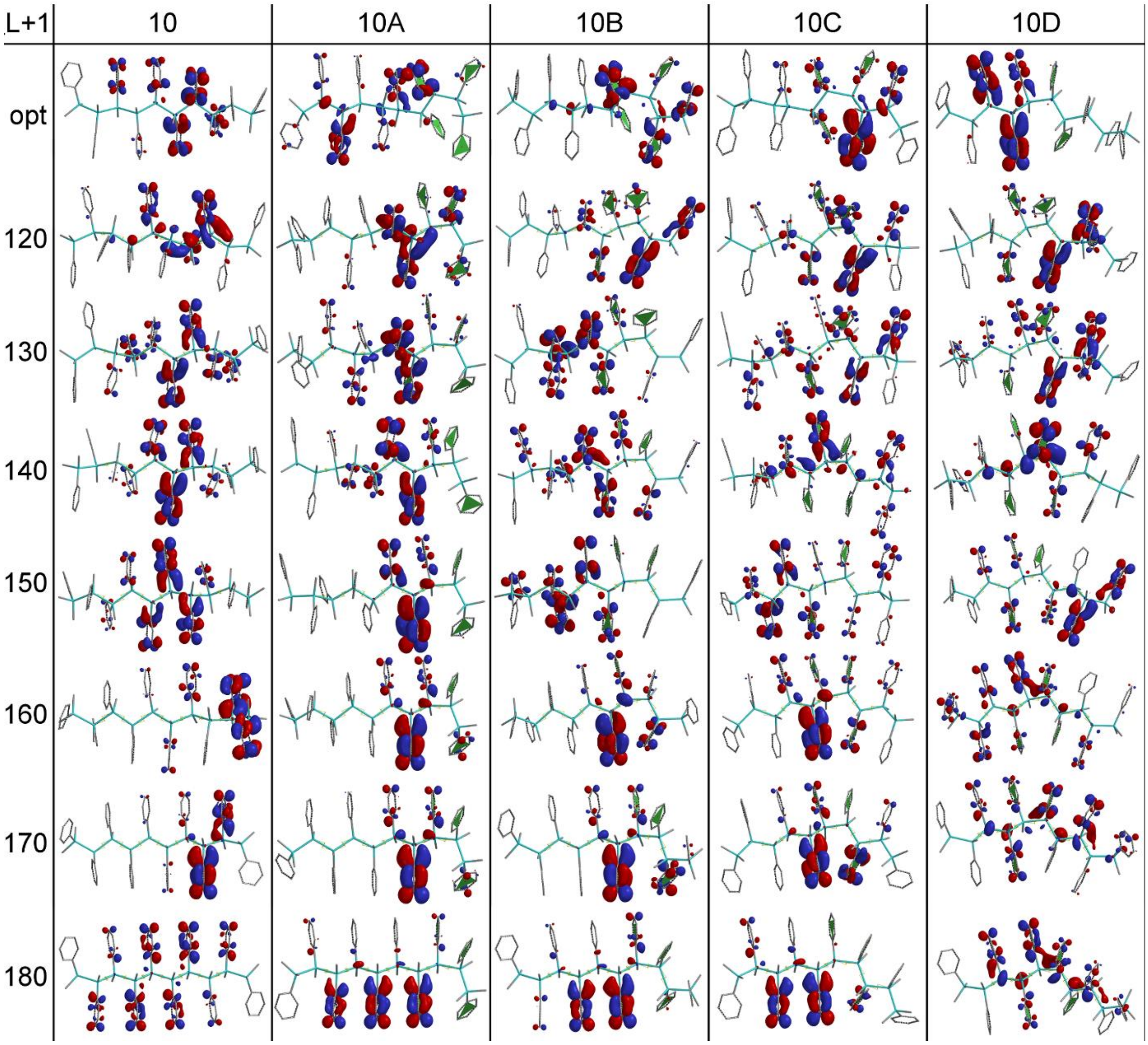

Figure S4. Kohn-Sham orbitals distribution: LUMO+1.
