# Supplementary material for: Effect of backbone conformation and its defects on electronic properties and assessment of the stabilizing role of π–π interactions in aryl substituted polysilylenes studied by DFT on deca[methyl(phenyl)silylene]s
Source: Chem Cent J. 2016 May 5;10:28. doi: 10.1186/s13065-016-0173-0 (PMC4858925; doi:10.1186/s13065-016-0173-0)
Supplement: Supplementary file 3 — 10.1186/s13065-016-0173-0 Contains a table with information on excitation process of all studied deca[methyl(phenyl)silylene]s in various backbone conformations and with an introduced kink in the chain. Backbone dihedral angles altered from 120° to 180° and the kink position altered from the edge of chain (A) to the centre part of chain (D). [file 13065_2016_173_MOESM3_ESM.pdf]

Effect of backbone conformation and its defects on electronic properties and assessment of the stabilizing role of π-π interactions in aryl substituted polysilylenes studied by DFT on deca[methyl(phenyl)silylene]s

Barbora Hanulikova, Ivo Kuritka, Pavel Urbanek*

Centre of Polymer Systems, Tomas Bata University in Zlín, trida Tomase Bati 5678, Zlin, 76001, Czech Republic

*Corresponding author, email: hanulikova@cps.utb.cz

Additional data file 3

Information on excitation process of all studied deca[methyl(phenyl)silylene]s in various backbone conformations and with an introduced kink in the chain. Backbone dihedral angles altered from **120°** to **180°** and the kink position altered from the edge of chain (**A**) to the centre part of chain (**D**).

Table S1 Description of excitation process for all OMPSi$_{10}$ (E – excitation energy, λ – wavelength of excitation, f – strength, TT – type of transition, Amp, - amplitude, P - percentage of allowed transition)

		10						10A						10B						10C						10D					
	ES	E[eV]	λ[nm]	f	TT	Amp.	P	E[eV]	λ[nm]	f	TT	Amp.	P	E[eV]	λ[nm]	f	TT	Amp.	P	E[eV]	λ[nm]	f	TT	Amp.	P	E[eV]	λ[nm]	f	TT	Amp.	P
120	7	4.1699	297.33	0.9208	H→L	0.9708	94	4.1817	296.49	0.7367	H→L	0.9698	94	4.2219	293.67	0.5475	H→L	0.9630	93	4.3223	286.85	0.3999	H→L<br>H→L+3	0.9155<br>-0.2189	84	4.3609	284.30	0.1020	H-1→L<br>H→L	0.2525<br>0.9225	85
	8	4.3142	287.38	0.0063	H→L+1<br>H→L+2	0.7706<br>-0.5664	59<br>32	4.3023	286.98	0.1196	H→L+1	0.9163	84	4.4512	278.54	0.0848	H→L+2<br>H→L+5<br>H→L+6	0.7875<br>-0.2444<br>-0.4179	42<br><br>17	4.4540	278.36	0.0337	H→L+1<br>H→L+2	0.7994<br>-0.491	64<br>24	4.4324	279.72	0.1283	H→L+1<br>H→L+2<br>H→L+3<br>H→L+4	-0.3810<br>0.6660<br>0.4427<br>-0.2208	15<br>44<br>20
	9	4.4253	280.17	0.0907	H→L+1<br>H→L+2<br>H→L+4	0.5406<br>0.7434<br>-0.2245	29<br>55	4.4556	278.27	0.0503	H→L+1<br>H→L+2	0.2332<br>0.9003	 81	4.4704	277.34	0.0553	H→L+1<br>H→L+2<br>H→L+3	0.7392<br>0.2224<br>0.5533	55<br><br>31	4.4643	277.73	0.0240	H→L+1<br>H→L+2<br>H→L+4<br>H→L+6<br>H→L+7	0.4287<br>0.5606<br>-0.427<br>0.3224<br>-0.2276	18<br>31<br>18<br>10	4.4468	278.82	0.0446	H→L+1<br>H→L+3<br>H→L+4	0.8252<br>0.2467<br>-0.3664	68
	10	4.4688	277.44	0.3522	H→L+2<br>H→L+4<br>H→L+5<br>H→L+7	0.2194<br>0.6132<br>0.5476<br>-0.2278	 38<br>30	4.5004	275.50	0.0855	H→L+3<br>H→L+4<br>H→L+5	0.7633<br>-0.2815<br>-0.4389	58<br><br>19	4.5266	273.90	0.0325	H→L+1<br>H→L+3<br>H→L+4	-0.4361<br>0.7513<br>-0.2777	19<br><br>56	4.5178	274.44	0.2476	H→L<br>H→L+3<br>H→L+4	0.2728<br>0.7543<br>0.3024	 <br>57	4.4888	276.21	0.3908	H-1→L<br>H-1→L+2<br>H→L+1<br>H→L+3<br>H→L+4<br>H→L+5	-0.4783<br>0.2126<br>0.2291<br>0.3529<br>0.4820<br>-0.3948	23<br><br><br>12<br>23<br>16
	11	4.4913	276.05	0.1210	H→L+3	0.9273	86	4.5304	273.67	0.1927	H-1→L<br>H→L+3<br>H→L+5	0.3130<br>0.5085<br>0.6882	 26<br>47	4.5708	271.25	0.0489	H-1→L<br>H-1→L+2<br>H→L+2<br>H→L+4<br>H→L+5<br>H→L+6	-0.2185<br>0.2615<br>0.2814<br>-0.3565<br>0.6467<br>0.3862	 <br><br>13<br>42<br>15	4.5727	271.14	0.1067	H-1→L<br>H→L+1<br>H→L+2<br>H→L+3<br>H→L+4<br>H→L+7	0.2944<br>-0.2323<br>-0.409<br>0.5209<br>-0.4584<br>-0.2281	 <br>17<br>27<br>21	4.5477	272.63	0.0268	H-1→L+1<br>H-1→L+2<br>H-1→L+3<br>H→L+2<br>H→L+3	-0.2725<br>-0.2713<br>-0.2421<br>-0.5121<br>0.6087	 <br><br>26<br>37
	12	4.5374	273.25	0.0315	H-1→L<br>H→L+4<br>H→L+5<br>H→L+6<br>H→L+7	-0.5002<br>-0.2328<br>0.3688<br>-0.6164<br>0.2245	25<br><br>14<br>38	4.5859	270.36	0.1505	H-1→L<br>H→L+3<br>H→L+4	-0.3923<br>0.2573<br>0.7832	15<br><br>41	4.6023	269.40	0.1685	H→L+1<br>H→L+4<br>H→L+5	-0.2187<br>0.8208<br>0.3430	 67	4.5901	270.11	0.0108	H-1→L<br>H-1→L+2<br>H-1→L+4<br>H→L+5<br>H→L+6	-0.219<br>0.2512<br>-0.4509<br>0.491<br>0.5019	 <br>20<br>24<br>25	4.5830	270.53	0.1727	H-1→L<br>H-1→L+2<br>H→L+3<br>H→L+4<br>H→L+7	0.5169<br>0.3450<br>0.3049<br>0.5074<br>-0.2762	27<br>12<br><br>26
130	7	4.0404	306.86	1.0192	H→L	0.9683	94	4.0899	303.15	0.9185	H→L	0.9672	94	4.1181	301.07	0.6299	H→L	0.9762	95	4.1951	295.54	0.4321	H→L<br>H→L+1	0.9194<br>0.2450	85	4.2885	289.11	0.1895	H→L<br>H→L+1	0.8842<br>0.2587	78
	8	4.2323	292.94	0.0083	H→L+1<br>H→L+2<br>H→L+3	-0.5027<br>0.5985<br>-0.5271	25<br>36<br>28	4.2698	290.37	0.0241	H→L+1<br>H→L+2<br>H→L+3	0.8657<br>-0.2269<br>-0.3067	75	4.3576	284.53	0.0683	H→L+1	0.9671	94	4.3716	283.61	0.1050	H→L<br>H→L+1<br>H→L+2<br>H→L+4	-0.2248<br>0.8485<br>-0.3407<br>-0.2411	 72	4.3477	285.17	0.1035	H-1→L<br>H→L+1	0.2434<br>0.8757	 77
	9	4.2900	289.01	0.0681	H→L+1<br>H→L+2<br>H→L+3	0.8319<br>0.4452<br>-0.2654	69<br>20	4.3571	284.56	0.0663	H→L+1<br>H→L+2<br>H→L+3<br>H→L+5	0.4466<br>0.5822<br>0.5879<br>0.2145	 34<br>35	4.4194	280.55	0.0356	H→L+2<br>H→L+3<br>H→L+6	-0.214<br>0.7860<br>0.3969	 62<br>16	4.4156	280.78	0.4154	H-1→L<br>H→L+2<br>H→L+3<br>H→L+4	0.3554<br>0.5969<br>-0.5138<br>-0.2336	13<br>36<br>26	4.3756	283.35	0.5470	H-1→L<br>H→L+1<br>H→L+3<br>H→L+4	0.2916<br>-0.2127<br>0.7573<br>0.3496	 <br>57<br>12
	10	4.3529	284.83	0.1812	H→L+2<br>H→L+3	0.5658<br>0.7664	32<br>59	4.4015	281.68	0.1510	H→L+2<br>H→L+3	0.7165<br>-0.6311	51<br>40	4.4505	278.58	0.0177	H→L+2<br>H→L+3<br>H→L+4	0.8198<br>0.3551<br>-0.2789	67	4.4320	279.74	0.0326	H→L+1<br>H→L+3<br>H→L+4	0.2407<br>-0.2739<br>0.8208	 <br>67	4.4343	279.60	0.1380	H-1→L+1<br>H→L+1<br>H→L+2<br>H→L+4<br>H→L+5	0.2947<br>-0.2182<br>0.6972<br>-0.3271<br>-0.4038	 <br>49<br>11<br>16
	11	4.4338	279.63	0.1472	H→L+6<br>H→L+7	0.8050<br>0.4103	65<br>17	4.4851	276.43	0.1279	H-1→L<br>H-1→L+3<br>H→L+3<br>H→L+4<br>H→L+5<br>H→L+6<br>H→L+8	0.2728<br>-0.2173<br>-0.2556<br>0.3649<br>0.6059<br>0.4074<br>0.2434	 <br><br>13<br>37<br>17	4.4933	275.93	0.1735	H→L+2<br>H→L+4	0.3339<br>0.8662	 75	4.4657	277.63	0.0830	H-1→L+1<br>H→L+1<br>H→L+2<br>H→L+3	-0.2166<br>0.2292<br>0.5878<br>0.6990	 <br>35<br>49	4.4846	276.46	0.0839	H→L+2<br>H→L+4<br>H→L+5	0.5104<br>0.4351<br>0.5835	26<br>19<br>34
	12	4.4415	279.15	0.0315	H→L+4	0.8894	79	4.5212	274.23	0.0965	H-1→L<br>H→L+4	-0.3342<br>0.6737	11<br>45	4.5215	274.21	0.0394	H-1→L<br>H→L+3	0.3491<br>-0.2719	12	4.5786	270.79	0.2045	H-1→L<br>H→L+4	-0.2168<br>-0.2567		4.5364	273.31	0.1587	H-1→L<br>H→L+3	-0.2295<br>-0.3369	 11

											H→L+5<br>H→L+7	-0.4703<br>-0.2980	22				H→L+5<br>H→L+6<br>H→L+8	0.4031<br>0.6513<br>0.2167	16<br>42				H→L+5<br>H→L+7	-0.7982<br>0.3008	64				H→L+4<br>H→L+5	0.6108<br>-0.5485	37<br>30
140	7	4.0366	307.15	0.9681	H→L	0.9671	94	4.0966	302.65	0.9709	H→L	0.9624	93	4.1520	298.42	0.7831	H→L<br>H→L+1	0.9411<br>0.2428	89	4.2310	293.04	0.6311	H→L<br>H→L+1	0.8261<br>-0.4610	68<br>21	4.2973	288.52	0.1880	H→L<br>H→L+1<br>H→L+2	-0.3215<br>0.6534<br>-0.5969	10<br>43<br>26
	8	4.2526	291.55	0.0811	H→L+1	-0.5027	96	4.2870	289.21	0.0285	H→L+1<br>H→L+2<br>H→L+3<br>H→L+6	0.7908<br>-0.2402<br>-0.4276<br>-0.2347	63<br><br>18	4.3303	286.31	0.0643	H→L<br>H→L+1<br>H→L+2<br>H→L+3<br>H→L+5	-0.2361<br>0.7776<br>0.2134<br>0.4253<br>-0.2263	 60<br><br>18	4.3338	286.08	0.0401	H→L<br>H→L+1<br>H→L+4	0.4822<br>0.7484<br>-0.3149	21<br>56	4.3004	288.31	0.5604	H-1→L<br>H→L<br>H→L+1	0.2427<br>0.7991<br>0.4371	 64<br>19
	9	4.2793	289.73	0.0199	H→L+2<br>H→L+3<br>H→L+4<br>H→L+5	0.6005<br>0.2427<br>0.4003<br>-0.5681	36<br><br>16<br>32	4.3275	286.5	0.0769	H→L+1<br>H→L+2<br>H→L+3<br>H→L+6	0.5600<br>0.5360<br>0.4691<br>0.5360	31<br>29<br>22	4.3762	283.31	0.0765	H→L+1<br>H→L+3<br>H→L+5	-0.5156<br>0.6432<br>-0.3652	27<br>41<br>13	4.3895	282.46	0.1659	H→L+2<br>H→L+3	0.8805<br>0.3008	78	4.3778	283.21	0.1411	H→L<br>H→L+1<br>H→L+2	-0.3094<br>0.5389<br>0.7439	 29<br>55
	10	4.3470	285.21	0.1146	H→L+2<br>H→L+3<br>H→L+4	-0.5596<br>0.6679<br>0.4028	31<br>45<br>16	4.3630	284.17	0.1080	H→L+2<br>H→L+3	0.7498<br>-0.5688	56<br>32	4.4163	280.74	0.1327	H→L+2<br>H→L+3<br>H→L+6	0.8935<br>-0.2630<br>-0.2122	80	4.4296	279.90	0.1486	H-1→L+1<br>H→L+1<br>H→L+3<br>H→L+4<br>H→L+5<br>H→L+6	-0.2155<br>0.3010<br>-0.5126<br>0.6258<br>-0.2840<br>-0.2168	 <br>26<br>39	4.4412	279.17	0.1018	H-1→L<br>H→L+3	-0.3439<br>0.8132	 66
	11	4.3816	282.96	0.0379	H→L+3<br>H→L+4<br>H→L+5<br>H→L+6<br>H→L+7	-0.2790<br>0.6712<br>0.4686<br>-0.3320<br>0.2166	 45<br>22<br><br>11	4.4281	279.99	0.0235	H→L+3<br>H→L+4<br>H→L+5<br>H→L+8	0.3017<br>0.6166<br>0.6279<br>-0.2155	 38<br>39	4.4812	276.68	0.0204	H→L+4<br>H→L+5	0.7721<br>0.5303	60<br>28	4.4557	278.26	0.1061	H→L+2<br>H→L+3<br>H→L+4<br>H→L+5<br>H→L+6	-0.2402<br>0.7225<br>0.2652<br>-0.3129<br>-0.2979	 52	4.4820	276.63	0.0722	H-1→L+3<br>H→L<br>H→L+4<br>H→L+6	-0.2913<br>0.2242<br>0.8312<br>-0.2545	 <br>69
	12	4.4062	281.38	0.0767	H→L+2<br>H→L+3<br>H→L+4<br>H→L+5<br>H→L+7	0.4349<br>0.5249<br>-0.3222<br>0.4370<br>0.3317	19<br>28<br>19<br><br>11	4.4709	277.31	0.0936	H→L+4<br>H→L+5<br>H→L+7<br>H→L+8	0.6655<br>-0.5329<br>-0.3005<br>0.2632	44<br>28	4.4976	275.67	0.1102	H→L+3<br>H→L+4<br>H→L+5<br>H→L+7	0.4541<br>-0.3997<br>0.6256<br>0.3741	21<br>16<br>39<br>14	4.5271	273.87	0.0325	H-1→L+4<br>H→L+4<br>H→L+5<br>H→L+7<br>H→L+8	-0.2496<br>0.3798<br>0.6941<br>-0.3059<br>-0.2718	 14<br>48	4.5155	274.57	0.2399	H-1→L+2<br>H→L+3<br>H→L+6<br>H→L+7	-0.5342<br>-0.3069<br>-0.2138<br>-0.6701	29<br><br><br>45
150	7	4.0773	304.08	1.2094	H→L	0.9615	92	4.1151	301.29	1.0888	H→L	0.9545	91	4.1795	296.65	0.9174	H→L	0.9509	90	4.2375	292.59	0.6312	H→L	0.9330	87	4.2880	289.14	0.3833	H→L	0.9266	86
	8	4.2852	289.33	0.0935	H→L+1<br>H→L+4	0.9168<br>0.2400	84	4.3040	288.07	0.0399	H→L+1<br>H→L+2	0.8721<br>-0.3835	76	4.345	285.35	0.0979	H→L+1<br>H→L+3	0.8761<br>0.4225	77<br>18	4.3653	284.02	0.1537	H→L+1<br>H→L+4	0.8507<br>0.4045	72<br>14	4.3475	285.18	0.2702	H→L+1<br>H→L+4	0.8612<br>-0.3167	74
	9	4.3095	287.70	0.0406	H→L+1<br>H→L+2<br>H→L+3<br>H→L+5	0.2326<br>0.8034<br>0.4102<br>-0.2295	 65<br>17	4.3159	287.27	0.0801	H→L+1<br>H→L+2<br>H→L+3	0.3804<br>0.8563<br>-0.2732	 73	4.3552	284.68	0.0605	H→L+2<br>H→L+4	0.8646<br>0.336	75	4.4059	281.41	0.0785	H→L+1<br>H→L+3<br>H→L+7	0.2581<br>0.9064<br>-0.2148	 82	4.4028	281.60	0.1508	H→L+2<br>H→L+3	0.7196<br>-0.6281	52<br>39
	10	4.3210	286.94	0.0105	H→L+1<br>H→L+3<br>H→L+4	-0.2687<br>-0.2746<br>0.8635	 <br>75	4.3562	284.61	0.0544	H→L+2<br>H→L+3<br>H→L+4<br>H→L+5	0.2571<br>0.7656<br>-0.3930<br>0.3211	 59<br>15<br>10	4.3858	282.70	0.0409	H→L+1<br>H→L+2<br>H→L+3<br>H→L+5<br>H→L+6	-0.3641<br>-0.3288<br>0.669<br>-0.2619<br>-0.3975	13<br>11<br>45<br><br>16	4.4169	280.70	0.0880	H→L+1<br>H→L+2<br>H→L+4<br>H→L+5<br>H→L+6	0.2518<br>0.5889<br>-0.5049<br>-0.2631<br>0.3889	 35<br>25<br><br>15	4.4425	279.09	0.0831	H→L<br>H→L+2<br>H→L+5	-0.2461<br>0.2682<br>0.8109	 <br>66
	11	4.3755	283.36	0.0185	H→L+3<br>H→L+6<br>H→L+7<br>H→L+8	0.3568<br>0.3287<br>-0.7493<br>0.2433	13<br>11<br>56	4.3872	282.60	0.0091	H→L+3<br>H→L+5<br>H→L+7	-0.3013<br>0.7096<br>0.3979	 50<br>16	4.4263	280.10	0.0339	H→L+2<br>H→L+3<br>H→L+4<br>H→L+6<br>H→L+7	-0.2345<br>-0.3732<br>0.6111<br>-0.4023<br>0.3775	 14<br>37<br>16<br>14	4.4246	280.22	0.0193	H→L<br>H→L+2<br>H→L+4<br>H→L+5<br>H→L+7	0.2277<br>0.5174<br>0.2700<br>0.6111<br>0.2551	 27<br><br>37	4.4500	278.61	0.1239	H-1→L+1<br>H→L+2<br>H→L+3<br>H→L+5<br>H→L+7	-0.2369<br>0.5651<br>0.6383<br>-0.2461<br>-0.2411	 32<br>41
	12	4.3857	282.70	0.1384	H→L+2<br>H→L+3<br>H→L+4<br>H→L+5<br>H→L+6	-0.2632<br>0.6887<br>0.2688<br>0.2807<br>-0.4201	 47<br><br><br>18	4.4166	280.72	0.0930	H→L+3<br>H→L+4<br>H→L+6<br>H→L+7	0.4340<br>0.7738<br>-0.2601<br>-0.2478	19<br>60	4.4867	276.34	0.0871	H→L+4<br>H→L+5<br>H→L+6	-0.3336<br>0.7994<br>-0.3134	 64	4.4836	276.52	0.0643	H-1→L+2<br>H→L+4<br>H→L+5<br>H→L+6	0.2689<br>-0.5537<br>0.5799<br>-0.3161	 31<br>34	4.4751	277.05	0.0384	H-1→L+3<br>H→L+1<br>H→L+4<br>H→L+7	-0.2385<br>0.3075<br>0.7777<br>0.3494	 <br>60<br>12
160	7	4.0690	304.70	1.3080	H→L	0.9746	95	4.1085	301.77	1.1393	H→L<br>H→L+4	0.9467<br>-0.2136	90	4.1771	296.82	1.0123	H→L	0.9270	86	4.2371	292.61	0.7266	H→L	0.9385	88	4.2879	289.15	0.5503	H→L	0.9117	83
	8	4.2986	288.43	0.1073	H→L+1<br>H→L+2<br>H→L+4	-0.5253<br>0.7842<br>-0.2508	28<br>61	4.2668	290.58	0.0590	H→L+1	0.9554	91	4.2902	288.99	0.0761	H→L+1	0.9402	88	4.3476	285.18	0.0908	H→L+1	0.9431	89	4.3569	284.57	0.2115	H→L+1<br>H→L+2<br>H→L+3<br>H→L+4	0.7663<br>0.3655<br>0.2424<br>-0.3489	59<br>13<br><br>12

	9	4.3175	287.17	0.0026	H→L+1<br>H→L+2<br>H→L+3<br>H→L+4	-0.542<br>-0.3314<br>0.6388<br>0.3049	29<br>11<br>41	4.3331	286.13	0.1405	H→L+2<br>H→L+3	0.9326<br>-0.2818	87	4.3372	285.86	0.1132	H→L+2<br>H→L+4	0.9291<br>-0.2416	86	4.3923	282.27	0.0715	H→L+2<br>H→L+4	0.8863<br>-0.3834	79	4.3925	282.27	0.1094	H→L+1<br>H→L+2	-0.4763<br>0.8250	23<br>68
	10	4.3521	284.88	0.0432	H→L+2<br>H→L+4<br>H→L+5	0.3096<br>0.7040<br>-0.5592	50<br>31	4.3801	283.06	0.0296	H→L+2<br>H→L+3<br>H→L+4<br>H→L+5	0.2452<br>0.7592<br>0.2958<br>-0.4715	58<br>22	4.386	282.68	0.0609	H→L+3<br>H→L+5	0.8705<br>-0.3677	76	4.4388	279.32	0.1367	H→L+2<br>H→L+3<br>H→L+4<br>H→L+5<br>H→L+6	0.2334<br>0.5320<br>0.6387<br>0.2126<br>-0.3461	28<br>41<br>12	4.4379	279.18	0.1575	H-1→L+1<br>H→L+1<br>H→L+3<br>H→L+5	-0.2637<br>0.2389<br>0.7432<br>-0.4539	55<br>21
	11	4.3660	283.97	0.1077	H→L+1<br>H→L+2<br>H→L+3	0.5836<br>0.3595<br>0.6731	34<br>13<br>45	4.3990	281.84	0.0126	H→L+3<br>H→L+4<br>H→L+6<br>H→L+7	-0.2620<br>0.5283<br>-0.5780<br>0.3901	28<br>33<br>15	4.4301	279.87	0.0403	H→L+4<br>H→L+5<br>H→L+6<br>H→L+7	0.6065<br>0.4211<br>-0.3727<br>0.4153	37<br>18<br>16<br>17	4.4862	276.36	0.0565	H→L+2<br>H→L+4<br>H→L+5	-0.2208<br>-0.2379<br>0.8220	68	4.4825	276.49	0.0579	H-1→L+3<br>H→L+1<br>H→L+2<br>H→L+4<br>H→L+5	-0.2506<br>0.2377<br>0.2728<br>0.7902<br>-0.3242	62
	12	4.4075	281.30	0.0335	H→L+3<br>H→L+4<br>H→L+5<br>H→L+6	-0.2263<br>0.4313<br>0.6222<br>-0.4652	19<br>39<br>22	4.4350	279.55	0.0575	H→L+3<br>H→L+5<br>H→L+6	0.3927<br>0.7124<br>-0.4113	15<br>51<br>17	4.4511	278.55	0.0574	H→L+3<br>H→L+4<br>H→L+5<br>H→L+8	-0.2419<br>0.62<br>-0.5161<br>-0.3133	38<br>27	4.4882	276.25	0.1032	H-1→L+3<br>H→L+3<br>H→L+4	-0.3185<br>0.5811<br>-0.4973	10<br>34<br>25	4.4496	275.77	0.1637	H-1→L+2<br>H→L+3<br>H→L+4<br>H→L+5<br>H→L+7	-0.2597<br>0.4958<br>0.2174<br>0.6389<br>0.3549	25<br>41<br>19
**170**	7	3.9203	316.26	1.3262	H→L	0.9783	96	3.9892	310.80	1.1801	H→L	0.9755	90	4.0856	303.47	1.0427	H→L	0.9634	93	4.1556	298.35	0.9813	H→L	0.9607	92	4.2313	293.02	1.0527	H→L	0.9305	87
	8	4.2896	289.03	0.0165	H→L+1<br>H→L+5	0.9282<br>-0.2219	86	4.2772	289.87	0.0361	H→L+1	0.9658	93	4.2876	289.17	0.0328	H→L+1	0.9600	92	4.3055	287.97	0.0143	H→L+1<br>H→L+2	0.8845<br>-0.3501	78	4.2952	288.66	0.0720	H→L+1	0.9493	90
	9	4.3491	285.08	0.0173	H→L+1<br>H→L+2<br>H→L+3	-0.2255<br>0.8013<br>-0.4430	66<br>20	4.3577	284.52	0.0225	H→L+2<br>H→L+3<br>H→L+5	0.8819<br>0.3140<br>-0.2494	78	4.3746	283.41	0.0277	H→L+2<br>H→L+3<br>H→L+6	0.8923<br>0.2406<br>-0.2680	80	4.3532	284.81	0.2360	H→L+1<br>H→L+2	0.3776<br>0.8755	77	4.3749	283.40	0.1273	H→L+2	0.9653	93
	10	4.3870	282.62	0.0074	H→L+2<br>H→L+3<br>H→L+5<br>H→L+6	0.2923<br>0.5316<br>-0.5292<br>-0.4996	28<br>28<br>25	4.4146	280.85	0.0878	H→L+2<br>H→L+3<br>H→L+4<br>H→L+5	-0.3395<br>0.5907<br>0.4089<br>-0.5420	12<br>35<br>17<br>29	4.4284	279.98	0.1243	H→L+3<br>H→L+4	0.5882<br>0.7270	35<br>53	4.4518	278.50	0.1080	H→L+3<br>H→L+4<br>H→L+5	0.7913<br>-0.4246<br>0.2183	63<br>18	4.4215	280.41	0.1749	H-1→L+1<br>H→L+3<br>H→L+7	0.3715<br>0.8263<br>-0.2214	14<br>68
	11	4.4094	281.18	0.1487	H→L+4	0.9292	86	4.4443	278.97	0.0220	H→L+4<br>H→L+5<br>H→L+6	0.6651<br>0.3540<br>-0.5026	44<br>13<br>25	4.4554	278.28	0.0439	H→L+2<br>H→L+3<br>H→L+4<br>H→L+5<br>H→L+7	-0.2877<br>0.6730<br>-0.5276<br>0.2809<br>-0.2391	45<br>28	4.4898	276.15	0.0896	H-1→L+1<br>H→L+4<br>H→L+5	0.2437<br>0.5962<br>0.6709	36<br>45	4.4846	276.46	0.0903	H-1→L<br>H→L+5	0.4067<br>0.8119	17<br>66
	12	4.4344	279.60	0.0425	H-1→L<br>H→L+2<br>H→L+3<br>H→L+5<br>H→L+7	-0.4415<br>0.3422<br>0.4551<br>0.3518<br>0.5031	19<br>21<br>12<br>25	4.4614	277.90	0.0372	H-1→L<br>H→L+3<br>H→L+4<br>H→L+5<br>H→L+7	-0.3828<br>0.5157<br>-0.2537<br>0.5615<br>0.2386	15<br>27<br>32	4.4675	277.53	0.1107	H-1→L<br>H→L+4<br>H→L+5	-0.2260<br>0.3450<br>0.8257	48	4.5022	275.38	0.0841	H→L+3<br>H→L+4<br>H→L+5<br>H→L+6<br>H→L+7	-0.4297<br>-0.4672<br>0.4812<br>-0.3156<br>0.3104	18<br>22<br>23	4.4942	275.88	0.1044	H-1→L<br>H-1→L+2<br>H→L+4<br>H→L+6	-0.2359<br>-0.2490<br>0.7197<br>-0.4389	52<br>19
**180**	5	3.8389	322.96	1.4727	H→L	0.9809	96	3.9221	316.20	1.2672	H→L	0.9801	96	3.9927	310.53	1.1435	H→L	0.9756	95	4.0906	303.09	1.0675	H→L	0.9684	94	4.1795	296.67	1.1367	H→L	0.9439	89
	8	4.2288	293.19	0.0000	H→L+1	0.9769	95	4.2608	291.04	0.0119	H→L+1	0.9795	96	4.2787	289.77	0.0084	H→L+1	0.9798	96	4.2826	290.86	0.0192	H→L+1	0.9667	93	4.2835	289.44	0.0892	H→L+1	0.9452	89
	9	4.2369	292.63	0.007	H→L+2	0.9754	95	4.3114	287.62	0.0019	H→L+2	0.9667	94	4.3089	287.74	0.0101	H→L+2	0.9740	95	4.3342	286.06	0.0606	H→L+2	0.9592	92	4.3807	283.02	0.0155	H→L+2	0.9226	85
	10	4.3740	283.46	0.0000	H-1→L<br>H→L+3	0.2713<br>0.9317	87	4.4239	280.25	0.0037	H-1→L<br>H→L+3<br>H→L+4	0.2308<br>-0.4964<br>0.7745	25<br>60	4.4270	280.07	0.0833	H-1→L<br>H→L+3	0.5902<br>0.7333	35<br>54	4.4129	280.96	0.0242	H-1→L<br>H-1→L+1<br>H→L+3	0.3421<br>-0.2280<br>0.8370	70	4.4016	281.68	0.1499	H-1→L+1<br>H→L+3	-0.3317<br>0.8523	73
	11	4.3854	282.72	0.0000	H-1→L<br>H→L+3<br>H→L+4	0.8783<br>-0.2451<br>-0.2868	77	4.4647	277.74	0.0085	H-1→L<br>H→L+3<br>H→L+4	0.6536<br>-0.4573<br>-0.4952	43<br>20<br>24	4.4850	276.44	0.0142	H→L+4<br>H→L+5<br>H→L+6<br>H→L+7	0.7629<br>-0.3318<br>-0.3684<br>0.2151	58<br>11<br>14	4.4575	278.14	0.2283	H-1→L<br>H-1→L+2<br>H→L+4	0.3420<br>-0.3121<br>0.8057	65	4.4493	278.66	0.0817	H-1→L<br>H→L+4<br>H→L+5	0.4791<br>0.3952<br>0.6995	23<br>16<br>49
	12	4.3945	282.13	0.0133	H-1→L+1<br>H→L+4	0.2302<br>0.9318	87	4.4809	276.73	0.0015	H-1→L<br>H→L+3<br>H→L+5<br>H→L+7	0.3180<br>0.4804<br>0.6491<br>0.3121	24<br>42	4.4993	275.60	0.0158	H-1→L<br>H→L+3<br>H→L+4<br>H→L+5<br>H→L+7	0.4526<br>-0.3949<br>0.2359<br>0.6133<br>0.2316	20<br>16<br>38	4.4815	276.66	0.0890	H-1→L<br>H→L+3<br>H→L+4<br>H→L+5	0.7217<br>-0.3672<br>-0.3479<br>-0.2808	52<br>13<br>12	4.4674	277.53	0.1431	H-1→L<br>H-1→L+2<br>H→L+4<br>H→L+5	-0.2346<br>0.2188<br>0.8205<br>-0.2895	67
