# Supplementary material for: Effect of backbone conformation and its defects on electronic properties and assessment of the stabilizing role of π–π interactions in aryl substituted polysilylenes studied by DFT on deca[methyl(phenyl)silylene]s
Source: Chem Cent J. 2016 May 5;10:28. doi: 10.1186/s13065-016-0173-0 (PMC4858925; doi:10.1186/s13065-016-0173-0)
Supplement: Supplementary file 4 — 10.1186/s13065-016-0173-0 Molecular energies calculated for all studied deca[methyl(phenyl)silylene]s obtained with B3LYP, M06 and ωB97X-D functionals. [file 13065_2016_173_MOESM4_ESM.pdf]

Effect of backbone conformation and its defects on electronic properties and assessment of the stabilizing role of π-π interactions in aryl substituted polysilylenes studied by DFT on deca[methyl(phenyl)silylene]s

Barbora Hanulikova, Ivo Kuritka, Pavel Urbanek*

Centre of Polymer Systems, Tomas Bata University in Zlín, trida Tomase Bati 5678, Zlin, 76001, Czech Republic

*Corresponding author, email: hanulikova@cps.utb.cz

Additional data file 4

Molecular energies calculated for all studied deca[methyl(phenyl)silylene]s obtained with B3LYP, M06 and ωB97X-D functionals.

Table S2 Molecular energies of all studied OMPSi$_{10}$ calculated with B3LYP, M06 and ωB97X-D functionals (ω – dihedral angle).

ω [°]	B3LYP [eV]	M06 [eV]	wB97X-D [eV]
	**10A**		
**120**	-154849.900	-154885.444	-154925.534
**130**	-154850.264	-154885.934	-154925.955
**140**	-154850.668	-154886.582	-154926.581
**150**	-154850.899	-154886.857	-154926.870
**160**	-154850.955	-154887.013	-154927.033
**170**	-154850.808	-154886.859	-154926.867
**180**	-154850.712	-154886.446	-154926.495
**opt**	-154850.980	-154886.893	-154926.909
	**10B**		
**120**	-154850.087	-154885.747	-154925.820
**130**	-154850.322	-154886.147	-154926.159
**140**	-154850.636	-154886.502	-154926.538
**150**	-154850.820	-154886.728	-154926.738
**160**	-154850.904	-154886.924	-154926.926
**170**	-154850.852	-154886.905	-154926.896
**180**	-154850.759	-154886.588	-154926.615
**opt**	-154850.987	-154886.870	-154926.879
	**10C**		
**120**	-154850.119	-154885.794	-154925.886
**130**	-154850.327	-154886.087	-154926.102
**140**	-154850.649	-154886.576	-154926.583
**150**	-154850.829	-154886.750	-154926.787
**160**	-154850.896	-154886.912	-154926.929
**170**	-154850.813	-154886.798	-154926.791
**180**	-154850.757	-154886.568	-154926.581
**opt**	-154850.963	-154886.847	-154926.856
	**10D**		
**120**	-154850.127	-154885.762	-154925.836
**130**	-154850.316	-154886.052	-154926.086
**140**	-154850.649	-154886.566	-154926.596
**150**	-154850.831	-154886.755	-154926.779
**160**	-154850.891	-154886.908	-154926.930
**170**	-154850.809	-154886.777	-154926.760
**180**	-154850.755	-154886.601	-154926.642
**opt**	-154850.949	-154886.887	-154926.904
	**10**		
**120**	-154849.736	-154885.131	-154925.221
**130**	-154850.157	-154885.747	-154925.760
**140**	-154850.677	-154886.635	-154926.634
**150**	-154850.928	-154886.866	-154926.873
**160**	-154850.945	-154887.008	-154926.995
**170**	-154850.807	-154886.921	-154926.892
**180**	-154850.676	-154886.416	-154926.472
**opt**	-154850.896	-154886.890	-154926.871
